# Optimized double-digest genotyping by sequencing (ddGBS) method with high-density SNP markers and high genotyping accuracy for chickens

**DOI:** 10.1371/journal.pone.0179073

**Published:** 2017-06-09

**Authors:** Yuzhe Wang, Xuemin Cao, Yiqiang Zhao, Jing Fei, Xiaoxiang Hu, Ning Li

**Affiliations:** 1 State Key Laboratories of Agro-biotechnology, College of Biological Science, China Agricultural University, Beijing, China; 2 National Engineering Laboratory for Animal Breeding, China Agricultural University, Beijing, China; Xiamen University, CHINA

## Abstract

High-density single nucleotide polymorphism (SNP) markers are crucial to improve the resolution and accuracy of genome-wide association study (GWAS) and genomic selection (GS). Numerous approaches, including whole genome sequencing, genome sampling sequencing, and SNP chips are able to discover or genotype markers at different densities and costs. Achieving an optimal balance between sequencing resolution and budgets, especially in large-scale population genetics research, constitutes a major challenge. Here, we performed improved double-enzyme digestion genotyping by sequencing (ddGBS) on chicken. We evaluated eight double-enzyme digestion combinations, and *Eco*R I- *Mse* I was chosen as the optimal combination for the chicken genome. We firstly proposed that two parameters, optimal read-count point (ORP) and saturated read-count point (SRP), could be utilized to determine the optimal sequencing volume. A total of 291,772 high-density SNPs from 824 animals were identified. By validation using the SNP chip, we found that the consistency between ddGBS data and the SNP chip is over 99%. The approach that we developed in chickens, which is high-quality, high-density, cost-effective (300 K, $30/sample), and time-saving (within 48 h), will have broad applications in animal breeding programs.

## Introduction

Genetic markers, as material for genetic research, have evolved from early restriction fragment length polymorphisms (RFLPs), amplified fragment length polymorphisms (AFLPs), and simple sequence repeats (SSRs) to currently widely-used SNP markers. Tremendous advances in genome-wide genotyping approaches have revolutionized the fields of population genetics and molecular breeding analysis [1]. Many different genotyping methods (such as whole genome sequencing, genome sampling sequencing, and SNP chips) have been developed, that vary in terms of marker density and cost. Since higher density leads to greater resolution but higher cost, achieving an optimal balance between the two constitutes a major challenge [2].

For research on population genetics, genotyping via whole genome sequencing is currently prohibitively expensive and technically unnecessary [3]. SNP chips, such as 600K oligonucleotide chicken arrays (Affymetrix, Inc., Santa Clara, CA, U.S.A.) [4] and 60K BeadArray microarrays (Illumina, Inc., San Diego, CA, U.S.A.) [5], are substantially less expensive, but possess limitations, such as: 1) less representative of Chinese local breeds; 2) inability to detect novel SNPs; and 3) applicable only to small-scale studies. However, the Reduced-Representation Genome Sequencing (RRGS) method has been recently developed [6–8], which refers to a group of various technologies with the principle of utilizing restriction enzyme digestion to reduce the loci to be sequenced. At present, numerous related methods are proposed, including restriction-site-associated DNA sequencing (RAD-seq) [9], genotyping by sequencing (GBS) [10], reduced-representation libraries (RRLs) [11], complexity reduction of polymorphic sequences (CRoPS) [12], their improved versions [13–16], etc. These RRGS methods are widely applied in animal, plant, and microorganism research [17–19].

Regarding chickens, RRGS approaches are widely employed. Kerstens et al. investigated genome-wide structure variations (SVs) by constructing reduced representation libraries (RRLs) of the chicken genome [20]. They identified hundreds of shared and divergent SVs in different layer and broiler lines. Zhai et al. discovered 75 K SNPs from 72 individuals, and 28 K SNPs were identified as candidates for 16 chicken breeds using the RAD-seq method [21]. Liao et al. further applied the genotyping by genome reducing and sequencing (GGRS) method in chickens, and identified 91 K SNPs from 252 individuals with lower cost [16]. In addition, Fábio et al. identified 134 K SNPs by optimizing the CornellGBS procedure [22]. For the researches above, single-enzyme (*Alu* I, *Hin*d III, *Ava* II, *Pst* I, respectively) was used for preparing sequencing libraries, under the guidance of the choosing of enzyme by either *in silico* digestion or extant literature [16,21,22]. However, the method of single-enzyme digestion might introduce some problematic issues, such as decreased sequencing quality caused by a high proportion of short fragments and inconsistency in the read counts per individual [16,22]. A meaningful diversification of GBS/RAD methods constituted the introduction of two enzymes. Some studies demonstrated that double-enzyme digestion generates more consistent results among different individuals than single-enzyme digestion [14,23]. However, to the best of our knowledge, the double-enzyme method has not yet been applied in chickens.

The required SNP marker density was determined by the extent of linkage disequilibrium (LD) in the experimental population. Previous studies have shown that the extent of LD varied significantly across different chicken breeds [24–26]. Generally, a minimum of 100 K SNPs are required to infer LD and haplotype information for the whole chicken genome [27]. For genome-wide association study (GWAS) and genomic selection (GS), a higher marker density is needed to increase resolution and accuracy, especially for populations with a low level of LD, such as advanced intercross lines (AILs) [28,29].

In this paper, we systematically evaluated the effects of various restriction enzymes and their combinations on the chicken genome. A nine-generation advanced intercross population was used to examine the ddGBS output. Our results showed that the *Eco*R I- *Mse* I combination was most suitable for chicken-GBS analysis. We proposed to use two parameters, optimal read-count point (ORP) and saturated read-count point (SRP), to determine the optimal sequencing volume. With an average sequencing depth of 10×, approximately 300 K SNP markers could be discovered with the *Eco*R I- *Mse* I combination.

Many RRGS adopted low-depth sequencing and imputation strategies. The common problem of these methods is high error rates in distinguishing heterozygous and homozygous individuals [8,30]. In this study, we validated the accuracy of genotyping utilizing various sequencing depth filter conditions by comparing the results to Illumina Chicken 60K BeadChip. Overall, we developed an optimized double-digest genotyping by sequencing (ddGBS) method with high-density SNP markers and high genotyping accuracy for chickens. Our experimental procedure could be applied to any other species.

## Materials and methods

### Ethics statement

All methods were carried out in accordance with relevant guidelines and regulations. All experimental protocols were approved by the Animal Welfare Committee of Agro-biotechnology of China Agricultural University. All animals used in this study were cared for and experimented on according to the requirements of the Animal Welfare Committee of Agro-biotechnology of China Agricultural University with the approval SKLAB-2014-06-07.

### Experimental population and sample preparation

We aim to assess ddGBS performance in a population with a low level of LD. A nine-generation advanced intercross population was established from two divergent chicken lines, High Quality chicken Line A (HQLA), a broiler line bred by Guangdong Wiz Agricultural Science and Technology, Co. (Guangzhou, China), and Huiyang Beard chicken (HB), a native Chinese meat-type breed. The F0—F_2_ cross has been described in detail by Sheng et al. [31]. After F_2_ generation, the population was bred by random mating. In total, a set of animal material, consisting of 31 F_0_ individuals, 191 F_8_ animals, and 602 F_9_ progeny, was selected. DNA was extracted from EDTA-anticoagulated blood using the Qiagen DNeasy Blood and Tissue Kit according to the manufacturer’s instructions (Qiagen, Hilden, Germany).

### Pre-sequencing processing and evaluation

In this study, both *in silico* simulation and empirical evidence were considered in choosing the proper enzyme for the digestion of chicken genome. We employed seven single-enzyme digestions (*Eco*R I, *Hin*P1 I, *Ape*K I, *Pst* I, *Mse* I, *Msp* I, and *Bgl* II, including four-/five-/six-cutter enzymes and restriction enzymes resistant to *dam*, *dcm*, and *CpG* methylation, methylation-sensitive: *Eco*R I, *Hin*P1 I, and *Ape*K I; methylation-insensitive: *Pst* I, *Mse* I, *Msp* I, and *Bgl* II) and eight double-enzyme digestions (*Pst* I-*Mse* I, *Pst* I-*Ape*K I, *Eco*R I- *Mse* I, *Bgl* II- *Ape*K I, *Pst* I- *Msp* I, *Hin*P1 I- *Mse* I, *Hin*P1 I- *Ape*K I, and *Eco*R I- *Msp* I) in our experiment. *In silico* analysis were conducted with an in-house Perl script. The size distribution of enzyme digestion fragments was reported using R software. Enzyme digestion experiments for all enzyme digestion combinations were performed according to the enzyme manufacturer’s protocol (New England Biolabs, Ipswich, MA, U.S.A.), and the digesting time for each combination was either 2 h or 12 h.

We employed double-digest genotyping by sequencing (ddGBS) on three samples from the F_0_ generation. All DNA samples were diluted to 50 ng/μL, and 200 ng DNA was used for each digestion of the eight double-enzyme combinations according to the enzyme manufacturer’s instructions. We designed 24 barcode adapters (eight enzyme combinations × three samples, see S1 Table). Mixing proportions of the barcode adaptors (BAs) and common adapters (CAs) were determined according to the fragment counts resulted from the *in silico* analysis of each restriction enzyme combination (S1 Protocol). The barcode adaptors (BA) were linked to the reverse complementary sequences of the Enzyme I overhang, and the common adaptors (CA) were linked to the reverse complementary sequences of the Enzyme II overhang. Library size-selection was implemented by Agencourt^®^ AMPure^®^ XP Reagent (Beckman Coulter, Pasadena, CA, U.S.A.): 0.8× and 1.3× sample volume of Agencourt^®^ AMPure^®^ XP Reagent can remove most of the short fragments (< 300 bp) and long fragments (> 650 bp), respectively. Detailed library preparation procedures are provided in S1 Protocol.

We evaluated each double-enzyme digestion strategy based on the enzyme digestion fragment size, the fragment consistency index (FCI), the coefficient of variation of sequencing depth (per fragment) across three samples (CV_depth_), the number of SNPs, and the distribution uniformity of SNPs across the chromosomes. We also subsampled reads of each individual in different proportions (10%, 20%, 50%, 80%, and 100%), and evaluated the “optimal read-count point (ORP)” and the “saturated read-count point (SRP)” parameters for cost optimization. A detailed definition of the above technical terms was described in the “Terminology” section.

### *Eco*R I- *Mse* I library preparation

All DNA concentrations were normalized to 50 ng/μL. Samples were digested for 12 h at 37°C with *Eco*R I- *Mse* I (New England Biolabs, Ipswich, MA, U.S.A.) in 20 μL volume containing 4 μL DNA (200 ng), 1× CutSmart^®^ Buffer, 5U *Eco*R I, and 5U *Mse* I. The enzymes were then inactivated by heating at 65°C for 20 min, and the samples were cooled to 4°C. The barcode adaptor (EcoR-BA) binds to the *Eco*R I overhang, and the common adaptor (Mse-CA) matches the *Mse* I overhang. The 96 indexes at the 3’ end of the barcode adaptors were designed by the GBS Barcode Generator (http://www.deenabio.com/) and modified to allow for Illumina NextSeq500 sequencer (San Diego, CA, U.S.A.) (no barcodes begin with GG; S2 Table). Barcodes were modulated in length between six and nine bases to prevent a decrease of sequencing quality near the restriction sites. 5 μL anneal adapter mix (the ratio of the EcoR-BA and Mse-CA is 0.8:15 based on the predicted fragment counts obtained from *Eco*R I and *Mse* I, S3 Table) was ligated to 20 μL digestion products by T4 DNA ligase (Invitrogen, Carlsbad, CA, U.S.A.). The reaction was incubated at 22°C for 1 h, and inactivated at 65°C for 20 min. Considering the maximum reads per flow cell of the NextSeq500 sequencer and the ORP of *Eco*R I- *Mse* I, 96 ligation products were pooled together (one library). Agencourt^®^ AMPure^®^ beads (Beckman Coulter, Pasadena, CA, U.S.A.) were used for DNA fragment purification and size-selection. The PCR amplification reaction system contained 10 ng purified products, 50 μL Platinum^®^ PCR SuperMix High Fidelity (Thermo, MA, U.S.A.), and 25 pmol primers (S1 Table). The amplification cycling protocol was as follows: 95°C for 5 min; three steps of 95°C for 30 s, 62°C for 30 s, and 68°C for 30 s for 17 cycles; followed by a final extension at 72°C for 5 min. PCR products were also purified by Agencourt^®^ AMPure^®^ beads. The fragment sizes obtained by this method were approximately 300 bp-650 bp, and the fragment size of the highest proportion was 350 bp. The final library quality (concentration and fragment size distribution) was determined by Qubit2.0 Fluorometer (Thermo, MA, U.S.A.) and Agilent 2100 Bioanalyzer (Agilent, Santa Clara, CA, U.S.A.), respectively.

### Sequencing and data processing

All sequencing experiments were performed on the Illumina Nextseq500 Sequencer at the State Key Laboratory for Agro-biotechnology, China Agricultural University. BCL files as primary sequencing output were converted into FASTQ files using bcl2fastq2 conversion software (version 2.16.0). During the conversion step, we also masked and trimmed the sequencing adapter [32]. After the trimming step, the Illumina 91-bp single-end reads were subjected to a filtering process: at first, the reads that were polluted by the adapter sequence were deleted, and then the reads which contained more than 50% low quality bases or more than 5% N bases were removed. The quality control check report of filtered reads was generated by FastQC software (http://www.bioinformatics.babraham.ac.uk/projects/fastqc/). We used TASSEL GBS analysis pipeline (version 4.0) [11,33], in which reads were aligned to the chicken reference genome Gallus_gallus-4.0 (released 2011) using Bowtie2 [34]. All SNP filter options in TASSEL were "-c 3", the minimum number of times a tag must be present to be output; "-mnTCov 0.01", the minimum SNP call rate for a taxon to be included in the output; "-mnSCov 0.6", the minimum sample call rate for a SNP to be included in the output; and "-mnMAF 0.05", the minimum minor allele frequency. The raw SNP sites were filtered by VCFtools [35] according to the following parameters: 1) minor allele frequency (MAF) > 5%; 2) genotypes with a quality above 98 (GQ ≥ 98) and depth ≥ 5; 3) and only biallelic markers were retained. Ungenotyped markers were imputed using Beagle4.0 software [36] with the pedigree file of F_8_-F_9_ family relationships. To annotate mutations from the GBS output, we used the SNPEff program [37], with the chicken reference genome sequence and GTF annotation files downloaded from Ensembl (http://www.ensembl.org/info/data/ftp/index.html). The Circos software package (http://circos.ca/) [38] was utilized to visualize the distribution of fragments, GC islands, repeat regions, and SNPs in the chicken genome. The genome-wide LD pattern assessment was implemented using a squared allelic correlation coefficient (r^2^) against the distance between the SNPs. To visualize the LD pattern, the r^2^ values were plotted against the pair-wise SNP distances.

### Terminology

A “good barcode read” is a sequence read with a perfect match to one of the barcodes provided in a barcode file. A “tag” refers to a unique sequence (excluding the barcode) from one or more “good barcode reads”. A “fragment” is defined as a set of tags that align to the exact same genomic position and strand. The number of tags and fragments is counted by the output file of the TASSEL software [33]. The fragment consistency index (FCI) is defined as the average fragment count from three samples divided by the total fragment counts obtained from pools of three samples. The sequencing depth is calculated as the total good barcode read counts divided by the fragment counts. The CV_depth_ is calculated as the mean of sequencing depth (per fragment) across three samples divided by the standard deviation (SD). The SNP density is defined as SNP number divided by chromosome length. The CV_SNP density_ is calculated as the mean of SNP density (per chromosome) divided by the standard deviation (SD).

The sequencing cost per fragment unit is calculated by total sequencing cost against fragment counts. The optimal read-count point (ORP) is defined as the minimum sequencing cost per fragment unit. We also defined the saturated read-count point (SRP) as the minimum good barcode reads when reaching the maximum fragment counts.

## Results

### Screen the appropriate enzyme combinations for the chicken genome

**Fig 1:**
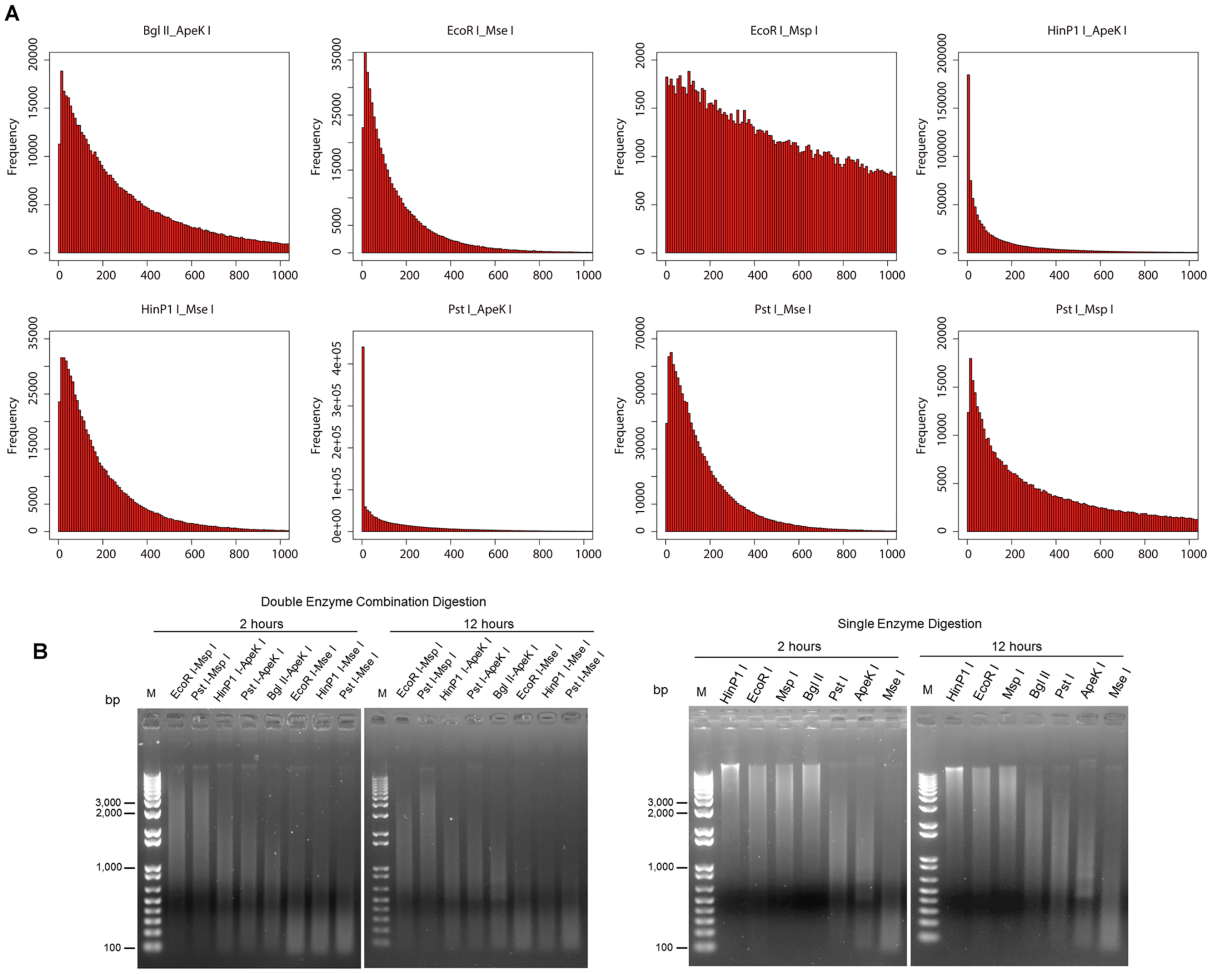
Results of both *in silico* analysis and empirical evidence of enzyme digestions. A) Fragment size distribution obtained by *in silico* digestion of the chicken genome with different double-enzyme combinations. B) Single-enzyme and double-enzyme digestion for 2 h or 12 h.

We performed a series of assessments for enzyme selection. The first parameter tested was fragment size. According to the predicted results from the *in silico* digestion, for every combination the majority of the predicted fragments were smaller than 500 bp (Fig 1A). In extreme cases, the *Pst* I- *Ape*K I and *Hin*P1 I- *Ape*K I combinations produced a high proportion of short fragments (< 100 bp). Although the size-selection step could theoretically filter out short fragments, it is difficult to remove them completely in practice. Specifically, too many short fragments will lower the quality of library construction and subsequent sequencing. In addition, by comparing the results of three methylation-sensitive enzymes (*EcoR* I, *Hin*P1 I, and *Ape*K I), we noticed that *Hin*P1 I (G/CGC) could not completely digest the chicken genomic DNA in 12 h, and the *Ape*K I (G/CWGC) digestion products exhibited a few discrete bands (Fig 1B). By contrast, the sizes of the *EcoR* I (G/AATTC) digestion product were appropriate (100 bp—1000 bp) and evenly distributed without discrete bands.

For a more accurate estimation, we carried out direct sequencing after digestion. We presented another four parameters, including the fragment consistency index (FCI), the coefficient of variation of sequencing depth (per fragment) across three samples (CV_depth_), the number of SNPs, and the distribution uniformity of SNPs across chromosomes. We prepared and sequenced 24 libraries of the eight double-enzyme digestions (three replicates for each combination, details are described in the Methods). Sequencing of all libraries produced a total of 365 million clean sequencing reads and 273 million good barcode reads, and all 24 barcode sequences were represented. The raw sequence reads were deposited in the SRA database (SRR3951559). A high FCI value represented high consistency and low level of missing data in different samples. We noticed that the fragment counts of the pooling sample (3-plex) was larger than the counts of each single sample at a high sequencing depth, indicating variance across different individuals (Table 1), which, in turn, might lead to missing data for the population. Moreover, we found that FCI was mainly determined by the types of combinations and independent of sequencing depth (FCI: 0.89, average depth: ~29× in *EcoR* I-*Mse* I; FCI: 0.64, average depth: ~36× in *Pst* I -*Msp* I; FCI: 0.58, average depth: ~16× in *Hin*P1 I- *Mse* I).

**Table 1:** Statistics of sequenced three samples from different combinations.

Enzyme	Individual	Good Barcode Reads	Fragments		Depth (×)	Tags	SNPs
			Number	Consistency Index (FCI)			
***Pst* I*—Mse* I**	***1***	31,092,630	974,736	-	31.90	1,191,540	-
	***2***	32,074,913	976,575		32.84	1,190,301	
	***3***	32,481,526	978,805		33.18	1,203,774	
	***3-plex***	95,649,069	1,247,742	0.7828	76.66	1,852,830	402,083
***Pst* I*—Ape*K I**	***1***	6,155,700	423,350	-	14.54	488,241	-
	***2***	14,690,702	562,974		26.09	679,210	
	***3***	15,964,678	577,803		27.63	700,684	
	***3-plex***	36,811,080	761,797	0.6844	48.32	1,043,308	195,960
***Eco*R I*—Mse* I**	***1***	8,191,164	351,880	-	23.28	409,007	-
	***2***	11,120,572	378,023		29.42	446,787	
	***3***	13,497,447	385,716		34.99	463,467	
	***3-plex***	32,809,183	414,294	0.8976	79.19	603,396	134,291
***Bgl* II*—Ape*K I**	***1***	11,435,477	356,686	-	32.06	425,531	-
	***2***	12,485,558	356,658		35.01	431,400	
	***3***	14,856,233	359,323		41.35	452,015	
	***3-plex***	38,777,268	436,503	0.8191	88.83	657,868	133,770
***Pst* I*—Msp* I**	***1***	10,777,226	313,086	-	34.42	418,533	-
	***2***	11,714,670	321,991		36.38	422,068	
	***3***	12,063,591	322,469		37.41	437,846	
	***3-plex***	34,555,487	498,114	0.6408	69.37	788,391	117,571
***Hin*P1 I*—Mse* I**	***1***	4,208,229	275,611	-	15.27	316,572	-
	***2***	4,663,311	289,381		16.11	323,745	
	***3***	4,786,013	292,617		16.36	331,145	
	***3-plex***	13,657,553	491,451	0.5817	27.79	629,468	94,724
***Hin*P1 I—*Ape*K I**	***1***	3,620,005	201,394	-	17.97	245,372	-
	***2***	3,515,377	194,682		18.06	238,182	
	***3***	4,661,900	218,302		21.36	295,108	
	***3-plex***	11,797,282	389,479	0.5258	30.28	533,246	71,751
***Eco*R I*—Msp* I**	***1***	2,635,952	75,407	-	34.96	93,221	-
	***2***	3,086,989	75,537		40.87	93,227	
	***3***	3,451,969	76,099		45.36	96,194	
	***3-plex***	9,174,910	96,527	0.7840	95.05	157,425	26,112

**Fig 2:**
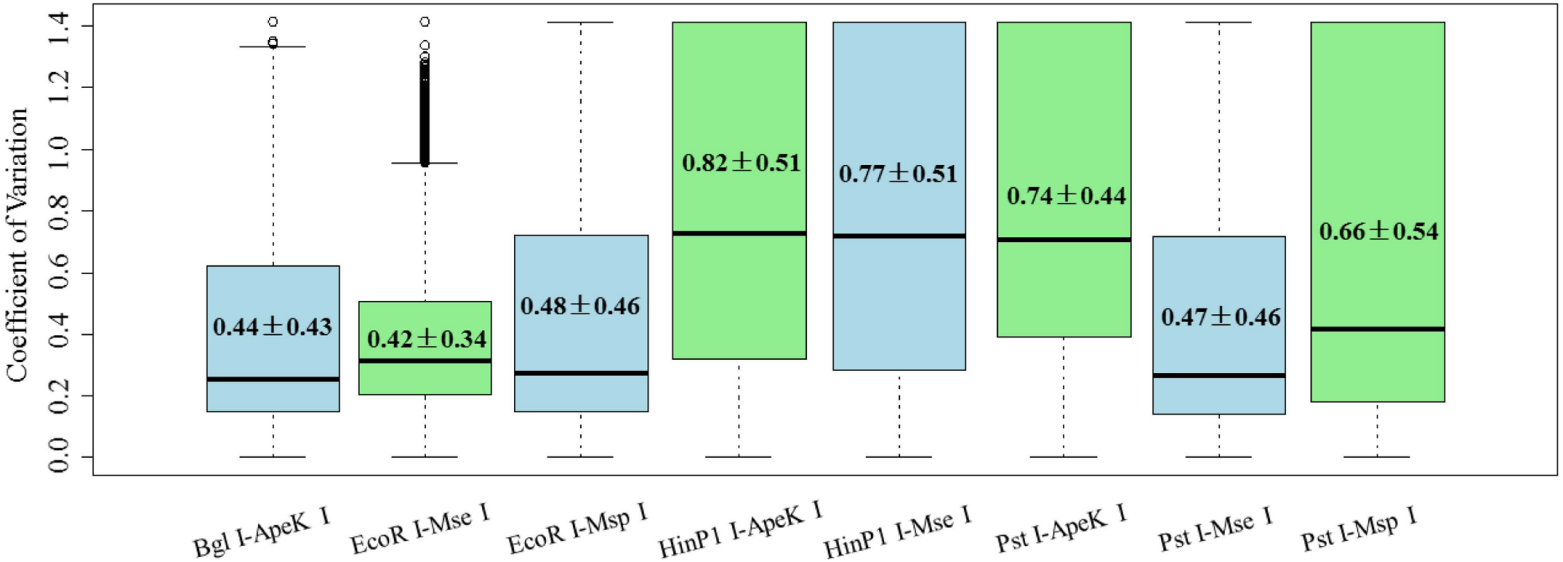
Distribution of CV_depth_ for each combination. Distribution of CV_depth_ for all fragments in each combination is displayed via boxplot. Lower and upper boundary lines of the boxes represent the 25%/75% quantile of CV_depth_, and the central lines indicate the median of the data. The upper and lower whiskers represent scores outside the middle 50%. The number in each box indicate the mean of CV_depth_ for all fragments±standard deviation (SD).

The consistency of sequencing depth (per fragment) across samples is also important because it is related to genotyping accuracy. We defined the CV_depth_ to evaluate the performance for each of the eight combinations. The distribution of CV_depth_ for all fragments in each combination was shown in Fig 2. *EcoR* I-*Mse* I had the lowest mean CV_depth_ across three individuals (0.42±0.34 (SD)) followed shortly after by *Bgl* II*—Ape*K I (0.44±0.43 (SD)). The highest mean CV_depth_ across three individuals occurred in *Hin*P1 I—*Ape*K I (0.82±0.51 (SD)) followed by *Hin*P1 I—*Mse* I (0.77±0.51 (SD)).

The number of SNPs was also critical, since too many (such as *Pst* I*—Mse* I) would increase sequencing cost, while too few (such as *Eco*R I*—Msp* I) would lower the resolution (Table 1). Another important factor for ddGBS was the distribution of SNPs per chromosome (Table 2). SNP density and coefficient of variation of SNP density (CV_SNP density_) across different chromosomes is shown in Table 2. The SNP discovered by *EcoR* I-*Mse* I and *Bgl* II-*Ape*K I was more evenly distributed across chromosomes with a CV_SNP density_ of 0.19 than other combinations. In contrast, the highest CV_SNP density_ was found in *Pst* I-*Mse* I (0.60) followed by *Hin*P1 I-*Ape*K I (0.59). Overall, the parameters among selected combinations were summarized in Table 3, and we concluded that the *Eco*R I- *Mse* I digestion was the optimal combination for the chicken ddGBS.

**Table 2:** Distribution of SNPs discovered from three individuals across chromosomes.

Chromosome		*Bgl* II*—Ape*K I		*Eco*R I*—Mse* I		*Eco*R I*—Msp* I		*Hin*P1 I—*Ape*K I		*Hin*P1 I*—Mse* I		*Pst* I*—Ape*K I		*Pst* I*–Mse* I		*Pst* I*–Msp* I	
Chr.	Size	SNPs	Density(N/Mb)	SNPs	Density(N/Mb)	SNPs	Density(N/Mb)	SNPs	Density(N/Mb)	SNPs	Density(N/Mb)	SNPs	Density(N/Mb)	SNPs	Density(N/Mb)	SNPs	Density(N/Mb)
chr1	195,276,750	26049	133	27690	142	4390	22	10356	53	15646	80	30930	158	64787	332	14385	74
chr2	148,809,762	20171	136	20915	141	3413	23	7653	51	11616	78	24048	162	50558	340	10548	71
chr3	110,447,801	16564	150	17220	156	2699	24	6266	57	9694	88	20977	190	42514	385	9148	83
chr4	90,216,835	11872	132	12425	138	2154	24	5508	61	7842	87	15280	169	32859	364	8322	92
chr5	59,580,361	7524	126	7751	130	1372	23	3766	63	4895	82	10879	183	21629	363	5482	92
chr6	34,951,654	5673	162	5490	157	1098	31	3019	86	4366	125	8760	251	17389	498	4578	131
chr7	36,245,040	4958	137	4994	138	947	26	2590	71	3801	105	7113	196	15051	415	3955	109
chr8	28,767,244	4058	141	4267	148	867	30	2391	83	2981	104	6403	223	12998	452	4147	144
chr9	23,441,680	4116	176	3997	171	874	37	2355	100	3110	133	6443	275	14076	600	4212	180
chr10	19,911,089	2667	134	2410	121	544	27	1954	98	2414	121	4470	224	9513	478	3509	176
chr11	19,401,079	2315	119	2666	137	495	26	1383	71	1965	101	3369	174	7627	393	2139	110
chr12	19,897,011	3199	161	2863	144	689	35	1994	100	2560	129	5725	288	11619	584	3847	193
chr13	17,760,035	2455	138	2246	126	659	37	2282	128	2677	151	4893	276	9649	543	3753	211
chr14	15,161,805	2341	154	1990	131	580	38	1897	125	2127	140	4774	315	9822	648	3850	254
chr15	12,656,803	1573	124	1420	112	421	33	1424	113	1525	120	3533	279	6470	511	2604	206
chr16	535,270	91	170	74	138	33	62	211	394	146	273	210	392	253	473	239	447
chr17	10,454,150	1843	176	1250	120	546	52	1832	175	1949	186	4483	429	8527	816	3950	378
chr18	11,219,875	1137	101	1043	93	331	30	1283	114	1434	128	2876	256	6021	537	2850	254
chr19	9,983,394	1788	179	1345	135	455	46	1521	152	1669	167	3794	380	7419	743	3138	314
chr20	14,302,601	2210	155	1927	135	599	42	1964	137	2247	157	5029	352	9690	677	3697	258
chr21	6,802,778	1031	152	856	126	265	39	941	138	997	147	2216	326	4276	629	1686	248
chr22	4,081,097	419	103	402	99	103	25	463	113	464	114	782	192	1760	431	754	185
chr23	5,723,239	948	166	865	151	425	74	1480	259	1311	229	2866	501	5701	996	2952	516
chr24	6,323,281	1061	168	762	121	327	52	1224	194	1468	232	2896	458	5512	872	2597	411
chr25	2,191,139	268	122	176	80	99	45	421	192	225	103	569	260	1109	506	810	370
chr26	5,329,985	830	156	553	104	262	49	1144	215	1015	190	2312	434	4473	839	2651	497
chr27	5,209,285	881	169	649	125	274	53	1216	233	939	180	2118	407	3974	763	2184	419
chr28	4,742,627	511	108	490	103	192	40	983	207	648	137	1767	373	3495	737	2156	455
chrZ	82,363,669	5016	61	5451	66	925	11	2105	26	2847	35	6292	76	12951	157	3228	39
chrW	1,248,174	201	161	104	83	74	59	125	100	146	117	153	123	361	289	200	160
Mean			142		126		37		130		135		277		546		236
CV_SNP density_			0.19		0.19		0.38		0.59		0.38		0.39		0.36		0.60
Total	1,003,035,513	133770		134291		26112		71751		94724		195960		402083		117571	

**Table 3:** Summary of parameters among different combinations.

Combinations	Fragment Size	Fragment Consistency Index (FCI)	Consistency of Sequencing Depth (assessed by CV_depth_)	The Number of SNPs (three individuals)	Uniformity of SNPs Distribution (assessed by CV_SNP density_ across chromosomes)
Bgl II—ApeK I	< 1000 bp	Medium	High	Medium	High
EcoR I—Mse I	< 1000 bp	High	High	Medium	High
EcoR I—Msp I	< 1000 bp	Medium	High	Low	Medium
HinP1 I—ApeK I	Large Proportion of Short Fragments	Low	Low	Low	Low
HinP1 I—Mse I	< 1000 bp	Low	Low	Low	Medium
Pst I—ApeK I	< 1000 bp	Medium	Low	Medium	Medium
Pst I—Mse I	Large Proportion of Short Fragments	Medium	High	High	Medium
Pst I—Msp I	< 1000 bp	Low	Medium	Medium	Low

### Determine the optimal level of sequencing depth

**Fig 3:**
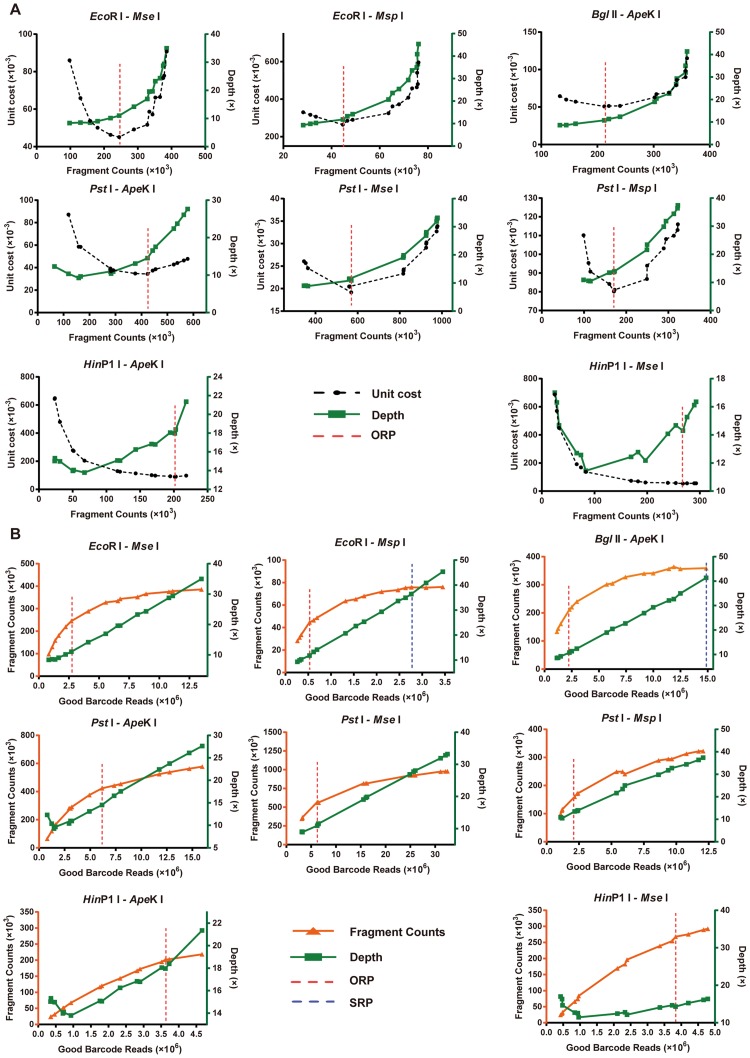
Relationship of ORP/SRP with good barcode reads for eight double-enzyme combinations. **A)** The function of unit sequencing cost of fragments was calculated by plotting sequencing depth versus fragment counts. The ORP was defined as the minimum value of the unit sequencing cost (the minimum value of the black-dashed line). **B)** The sequencing reads of three individuals were sampled at five thresholds (10%, 20%, 50%, 80%, and 100%, respectively). The sequencing depth (green) was equal to the good barcode read numbers divided by the fragment counts. The SRP was the corresponding good barcode reads when the slope of the fragment counts (orange curve) reduced to zero.

In order to obtain the optimal sequencing depth, we resampled a series of incremental subsets from total sequencing reads, and then investigated the relationship among the fragment counts, sequencing depth, and good barcode read counts. Different from whole genome sequencing, in which depth was calculated as the total length of the raw reads divided by the fixed total length of reference genome, in ddGBS the depth would be calculated as the total good barcode read counts divided by the fragment counts, which would increase with amount of sequencing until saturation. We estimated the two parameters: ORP (the minimum sequencing cost per fragment unit, Fig 3A) and SRP (the point at which there is a tangent line with zero slope for the fragment counts curve in Fig 3B) for all eight double-enzyme digestion libraries.

Fig 3A shows that the unit cost firstly decreased and then increased with the increasing of fragment counts. The unit cost fell to the lowest level (ORP) at approximately 10× sequencing depth in most combinations. Fig 3B shows how the fragment counts change as a function of good barcode reads. In this study, no SRPs were reached in most combinations, except *Eco*R I- *Msp* I and *Bgl* II- *Ape*K I, even though the sequencing depth was greater than 20× (Fig 3B). Theoretically, the fragment counts would be saturated when good barcode reads continued to increase, and saturation is expected to ensure consistency among different individuals. However, a typical GWAS/GS examines several hundred individuals. It is impractical to sequence all of the individuals to the saturation level (ranging in depths from 30× to 50×) for large populations (such as a family-based population with individuals of more than 100). One affordable design is to reduce the amount of sequencing appropriately and impute the missing genotypes. Taking the *Eco*R I- *Mse* I digestion as an example, at ORP, there were 2.7 million good barcode reads and approximately 270 K fragment counts for each sample, and the average sequencing depth was 10× for each fragment. Therefore, this compromise formula is not only highly precise, but also cost- effective.

### SNP discovery and distribution

A total of 827 samples (824 chickens, among which three individuals were duplicated) in AIL were used to construct the ddGBS libraries. 96-plex samples were sequenced in one lane according to the ORP of *Eco*R I- *Mse* I. The raw sequence reads were deposited in the SRA database (SRR5462540, SRR5462541, SRR5462542, SRR5462543, SRR5462544, SRR5462545, SRR5462546, SRR5462547, and SRR5462548). On average, 3.44 million good barcode reads were obtained for each sample, and the average sequence depth was approximately 10×. The coefficient of variation (CV) of read counts among individuals was 0.13 (S1 Fig), indicating good consistency of library preparation. The SNPs ranged from 220–270 K among individuals prior to imputation. After strict parameter filtering in the TASSEL-BEAGLE-GBS pipeline (including imputation), we identified 291,772 SNPs ultimately (average sequencing depth was 10× with no missing data), corresponding to 1 SNP per 3.68 Kb in the chicken genome (S2 Fig and S4 Table). It is worth noting that the marker density is higher than what was reported in previous studies in chickens [16,21,22]. Among all discovered SNPs, 102,304 (accounting for 35.06% of all SNPs; the distribution is shown in S4 Table) are novel to the NCBI chicken dbSNP database (data from ftp://ftp.ncbi.nih.gov/snp/organisms/chicken_9031/VCF/ on May 4, 2016). In addition, the markers were evenly distributed without interference from GC islands and repeat regions (S2 Fig). The majority of SNPs identified were located in intergenic regions (45.19%) or intronic regions (39.55%). The exonic regions contained only 1.37% of SNPs (Table 4), comprising 51.69% missense, 3.57% nonsense, and 44.74% silent mutations.

**Table 4:** Number of SNPs by region.

Type	Count	Percent (%)
**UPSTREAM**	18441	6.32
**UTR_3_PRIME**	2428	0.832
**EXONIC**	4005	1.373
--MISSENSE	2070	51.69
--NONSENSE	143	3.57
--SILENT	1792	44.74
**INTRONIC**	115399	39.551
**INTERGENIC**	131862	45.193
**UTR_5_PRIME**	298	0.102
**DOWNSTREAM**	18899	6.477
**Others**	440	0.15

### Genotyping accuracy evaluation

**Fig 4:**
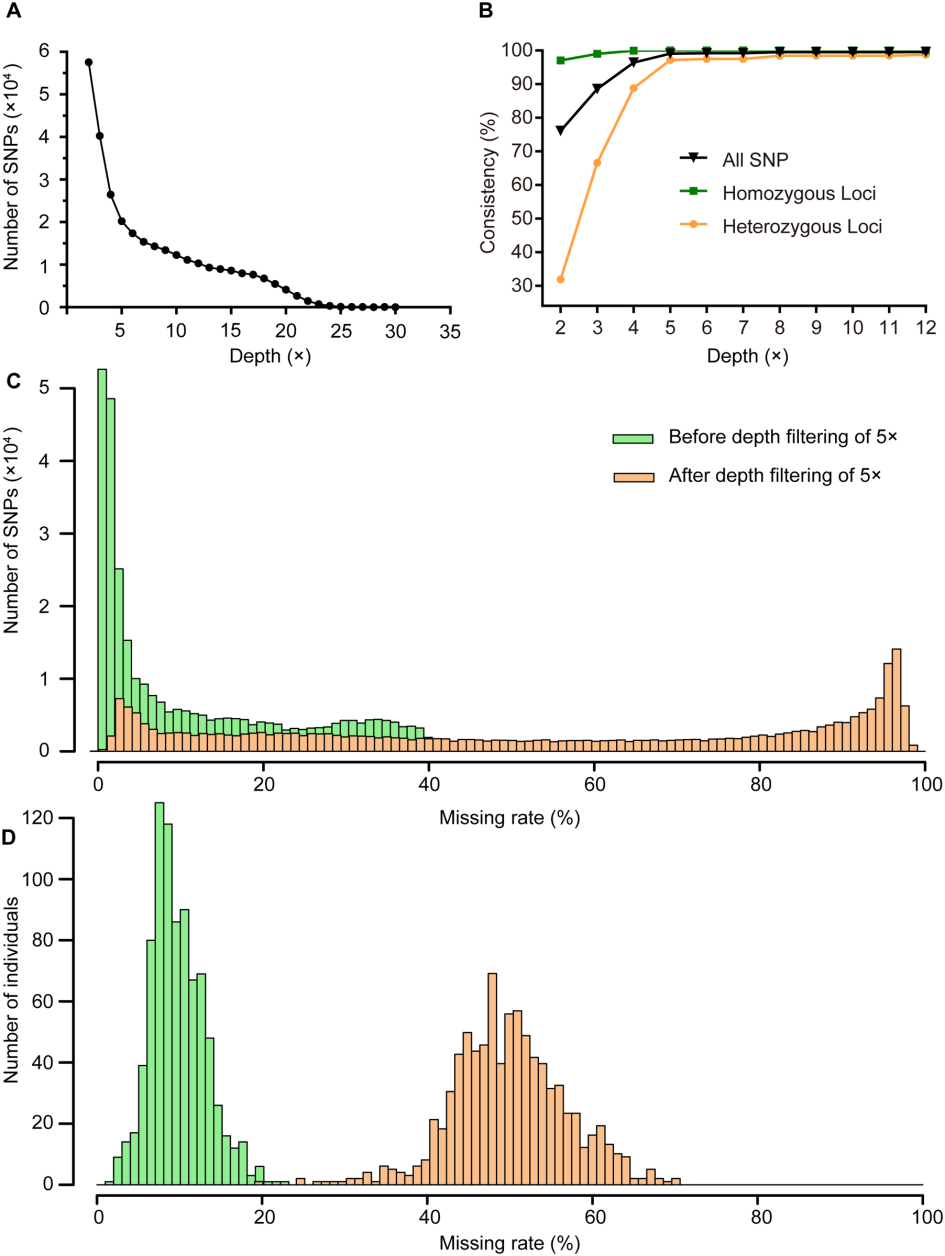
Genotyping accuracy evaluation according to different sequencing depths. **A)** Distribution of coverage depths for all SNPs. **B)** Consistency of ddGBS genotyping results compared with 60K BeadArray microarrays using various filter conditions (sequencing depth ranging from 2× to 12×). **C)** Comparison of the missing rates of all 292 K SNPs on a per-site basis before and after depth filtering of 5×. **D)** Comparison of the missing rates of all 824 samples on a per-individual basis before and after depth filtering of 5×.

The average sequencing depth of our experiments was 10×, with an abundance of low coverage SNP sites (Fig 4A). In order to guarantee high-quality genotyping of the founders prior to imputation, we filtered raw SNP data using sequencing depth and genotyping quality, as well as minor allele frequency. Sufficient depth at each locus is essential to accurately distinguish heterozygous and homozygous sites. To assess the accuracy of genotyping, Illumina 60K chicken BeadArray microarray data and GBS results from 22 same F_0_ individuals were compared. The correspondence between the two methods was evaluated at different depths ranging from 2× to 12×. When the sequencing depth reached 5×, the genotyping consistency for homozygous loci, heterozygous loci and total SNPs was 100%, 97.2%, and 99.1%, respectively (Fig 4B). The missing rates of SNPs with the 5× sequencing depth are shown in Fig 4C and 4D. After depth filtering of 5×, about 45.7% SNPs contained ≤ 50% missing genotypes (Fig 4C). The missing rates of most of the samples are between 40% and 60% (Fig 4D). We also performed two technique repeats for SNP calling for three samples, and found that reproducibility (in the case of the 5× filter condition) reached 98.5%, 98.2%, and 98.1%, respectively. Therefore, our results indicated that the genotyping results of our methods are highly reliable and accurate.

## Discussion

To design a ddGBS plan, multiple factors needed to be considered, including selection of enzyme combinations, optimization of library construction, sequencing depth of coverage, SNP density, and cost.

### Selection of enzyme combinations

The selection of enzymes constitutes one of the key steps in the GBS method, and is often neglected. In this study, we investigated five parameters obtained from *in silico*/ *in vitro* digestion or sequencing. We found that the result of *Hin*P1 I enzyme digestion was not consistent with its *in silico* simulation. Specifically, the fragments produced by *Hin*P1 I- *Mse* I and *Hin*P1 I- *Ape*K I were far fewer than the results of *in silico* simulation. This difference could be mainly due to DNA methylation in chicken genome. Indeed, some methylation sensitive enzymes cannot digest the genome completely, which will not only cause inconsistency with the predicted results, but also interferes with the reproducibility of downstream genotype calling among different individuals [8]. Therefore, a pre-assessment experiment is necessary when markers in a new species are to be developed.

The distribution of SNPs obtained by different enzymes was tested in this study. We noticed that GGA10-20 has two-fold more fragment density than the GGA1-10 for *Pst* I and *Msp* I *in silico* prediction (S3 Table), which may explain the result that the fragments generated from *Pst* I- *Msp* I were not evenly distributed between macrochromosomes and microchromosomes. An uneven distribution of SNPs may have hampered the construction of evenly-distributed genetic linkage maps. However, Fábio suggested that *Pst* I would be suitable for chicken methylation analysis since the microchromosomes are enriched for high CpG regions [22].

### Library construction process

There are two key points in library preparation that need to be addressed. First, we improved the original GBS approach described by Poland et al. in the size-selection step [23]. In previous study, 37% reads were discarded in the data processing step since the fragment size was too short (< 50 bp). Here, we removed long fragments (> 650 bp) by adding 1.3× sample volume of Agencourt^®^ AMPure^®^ XP Reagent, and removed short fragments (< 300 bp) by adding 0.8× sample volume of Agencourt^®^ AMPure^®^ XP Reagent (details are described in S1 Protocol). In our experience, magnetic bead purification was more convenient, and exhibited better consistency among different libraries compared to gel extraction.

Second, the accurate concentration of double-stranded DNA could improve the consistency of good barcode read numbers for each sample. De Donato reported that the read number per sample varied by 39% when 47 individuals were digested by *Pst* I [18]. Several other studies have also observed high CV in the number of different individuals (0.69 for 252-plex in chickens [16], 0.89 for 96-plex in *Drosophila* [39], etc.). Liao suggested that this should result from poor DNA quality, such as inaccurate quantification or contamination of DNAs with phenol/chloroform [16]. To ensure the uniformity of DNA concentration, high-molecular-weight DNA concentration was measured by Qubit2.0 prior to enzyme digestion in this study. Sequencing results showed that all of the 824 samples were well represented, and the CV of good barcode reads was 0.13 (S1 Fig), which was better than that achieved in previous studies [9,16,18,39,40]. Moreover, we noticed that the majority of missing rates of samples ranged from 40% - 60% (Fig 4D). This phenomenon may be attributed to the low CV of good barcode reads among samples.

### Characteristics of sequencing depth per site

The number of SNPs declined with the increase of minimum depth used for identifying SNPs (Fig 4A), which was similar to other studies that used the GBS method [41,42]. A possible cause for the distribution was the inconsistency in the depth per fragment. The number of fragments and tags (which refers to a unique sequence from one or more good barcode reads) was counted with the Tassel parameter “-c” of 3 [33], which required a tag to be presented at least three times to be reported. Thus, a number of fragments of low depth were discarded in single sample analysis, but still retained in 3-plex pooling sample analysis, which might be the main reason for FCI < 1 (Table 1). We also noticed the divergent FCI value from the eight combinations and its dependence of combination type rather than the sequencing depth. The possible reason is that methylation (such as *Hin*P1 I) or polymorphism disrupted a restriction site in varying extents among different samples [8]. Moreover, the difference in enzyme activity may affect the efficiency of enzyme digestion.

The accuracy of genotyping constitutes another key aspect of GBS technology. Currently, a typical GBS combines low-depth sequencing (for some inbred line of maize, the depth < 1×) and missing data imputation [43–45]. However, this strategy works better for populations with a low level of heterogeneity, such as recombinant inbred lines (RILs) rather than outcrossing populations. Genotyping errors in calling heterozygotes as homozygotes are quite common in GBS, either due to the low depth of reads or the incorrect read alignment resulting from paralogous regions. Our results showed that the 5× depth was the lowest depth for accurate SNP calls prior to the imputation step. Under this condition, the reliable SNPs of each individual were approximately 150 K, which was still higher than that in other studies [16,21]. The refined identity-by-descent (IBD) method implemented in Beagle 4.0 achieved a better performance [35].

### SNP density and cost

**Fig 5:**
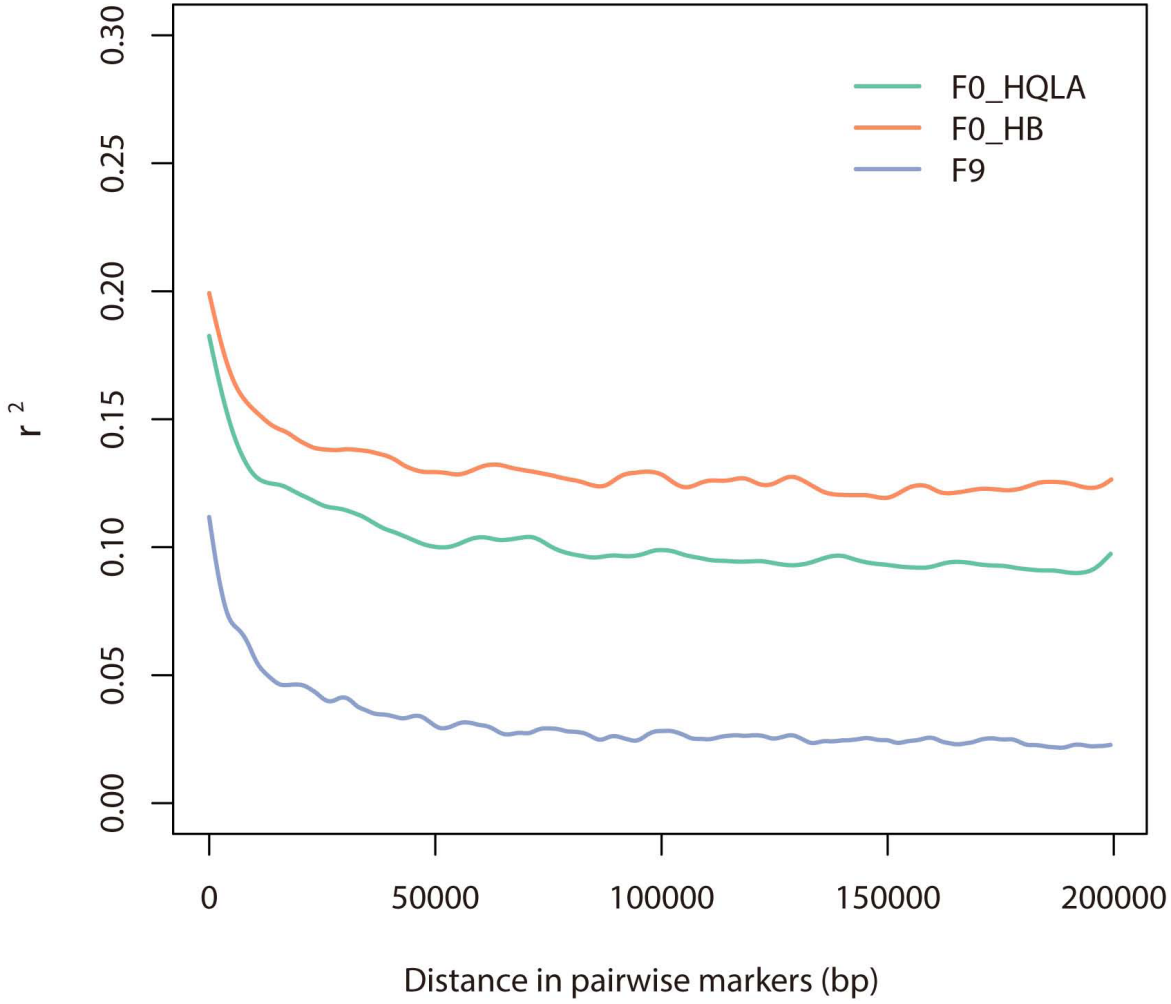
LD decay of the advanced intercross chicken lines. A squared allelic correlation coefficient (r^2^) against the distance between the SNPs in the F_0_ generation (HQLA was depicted as the green line, and HB was depicted as the red line) and F_9_ generation (the blue line).

In this study, we developed a high-density and accurate SNP genotyping method for chickens using *EcoR* I- *Mse* I. The SNP density was approximately 290 K, and some minichromosomes were not included. AIL population was commonly used for QTL fine-mapping in animal genetics [28,46–48]. Applying the SNP markers that we identified on our chicken population, we noticed that r^2^ in the F_9_ generation, which was r^2^_0.1_ = 3.1 Kb, was substantially lower than the F_0_ generation (r^2^_0.1_ > 50 Kb) (Fig 5). This suggested that, although eight generations of recombination decrease LD levels effectively, our marker density (SNP/3.68 Kb) can still capture almost all recombination events. Thus, it can greatly benefit the fine-mapping of the QTL locations and functional genes.

The preliminary experiment was introduced to determine the optimal enzyme combination (*EcoR* I- *Mse* I) and approximate scope of the sequencing depth (ORP for large-scale population and SRP for a small number of samples). Consequently, we achieved a balance between the density of SNPs and cost. This pre-assessment method is recommended for any novel species.

Reducing cost constitutes a primary aim of all reduced-representation genome sequencing methods. Thus, our method has been optimized for cost at almost every step. However, Illumina HiSeq X Ten pair-end 150 sequencing is now much more cost-effective than single-end sequencing using NextSeq500. Moreover, adopting pair-end sequencing would provide a better chance in SNP identification than single-end sequencing in this study. Currently, ddGBS costs $30 per sample (approximately 300 K SNPs/individual), and more than 65% of the expense comes from the sequencing step in our protocol. Therefore, decreases in the cost of GBS are expected with the rapid development of sequencing technology. For example, HiSeq X Ten systems can output 800–900 Gb data/2.6–3 billion reads in a single flow cell with a cost of $1,000 for 30× of the human genome, which would be more appropriate for large-scale population sequencing (http://www.illumina.com/systems/hiseq-x-sequencing-system/system.html). The RRGS process will be quickly standardized with the declining cost of sequencing and it will, together with SNP chips, continue to be a crucial method for genomics study. In addition, the combination of RRGS and other genome-wide sampling sequencing, such as RNA-seq or Targeted Re-sequencing, could effectively promote genetics and evolutionary studies. In conclusion, we present an accurate, high-density, and cost-effective genotyping method for chickens. Our method could facilitate functional gene mapping and molecular breeding of agricultural animals, and could easily be applied to any other species.

## Supporting information

S1 Fig Number of good barcoded reads per sample. The x-axis denotes the 824 samples, and the y-axis denotes the good barcoded reads. Sample ID number was sorted by the number of sequencing reads.
(PDF)

S2 Fig SNP and tag distribution across the chicken genome in 824 individual samples digested by *Eco*R I- *Mse* I. In total, 292 K SNPs were identified among all individuals. The genome characteristics and genome-wide distribution of restricted digest fragments are represented circularly. The exterior circle displays the lengths of the chromosomes. The four interior circles show the distribution of fragments (green), GC islands (orange), repeat regions (black), and SNPs (red) from outside to inside.
(PDF)

S1 Table PCR primers and 24 barcode sequences. (PDF)

S2 Table Barcode adaptor (BA) sequences (96-plex). (PDF)

S3 Table Number of predicted fragments obtained from seven enzymes and their distribution across the chromosomes. (PDF)

S4 Table Number of genes, SNPs, and novel SNPs discovered from ddGBS, 60K, and 600K SNP chips. (PDF)

S1 Protocol Double-digest genotyping by sequencing protocol. (PDF)
