# Supplementary material for: Optimized double-digest genotyping by sequencing (ddGBS) method with high-density SNP markers and high genotyping accuracy for chickens
Source: PLoS One. 2017 Jun 9;12(6):e0179073. doi: 10.1371/journal.pone.0179073 (PMC5466311; doi:10.1371/journal.pone.0179073)

**S1 Fig. Number of good barcoded reads per sample.** The x-axis denotes the 824 samples, and the y-axis denotes the good barcoded reads. Sample ID number was sorted by the number of sequencing reads.

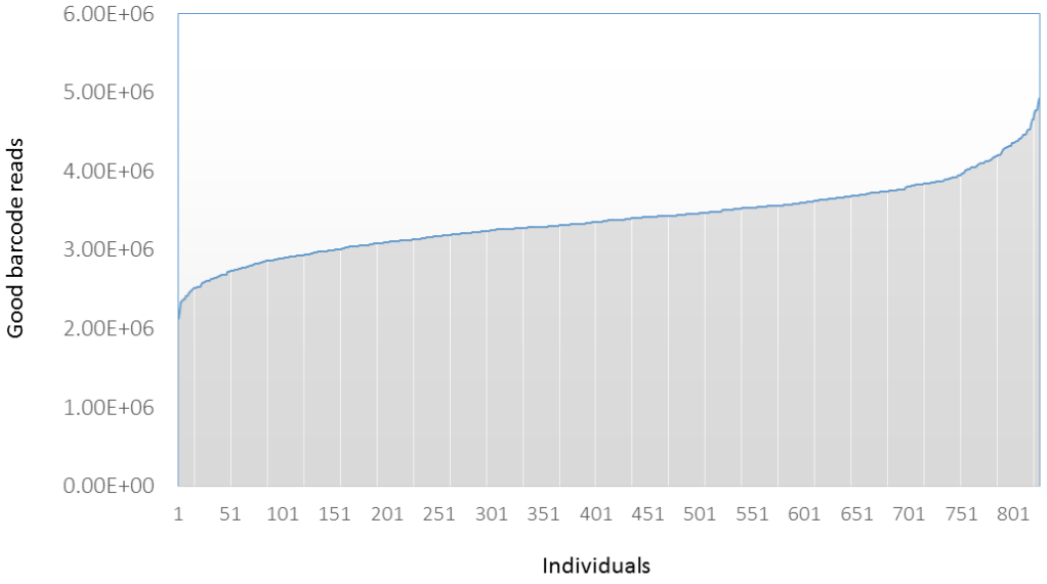

Supplement: S1 Fig — The x-axis denotes the 824 samples, and the y-axis denotes the good barcoded reads. Sample ID number was sorted by the number of sequencing reads. (PDF) [file pone.0179073.s001.pdf]
