# Supplementary material for: Optimized double-digest genotyping by sequencing (ddGBS) method with high-density SNP markers and high genotyping accuracy for chickens
Source: PLoS One. 2017 Jun 9;12(6):e0179073. doi: 10.1371/journal.pone.0179073 (PMC5466311; doi:10.1371/journal.pone.0179073)

S2 Fig. SNP and tag distribution across the chicken genome in 824 individual samples digested by *Eco*R I- *Mse* I. In total, 292 K SNPs were identified among all individuals. The genome characteristics and genome-wide distribution of restricted digest fragments are represented circularly. The exterior circle displays the lengths of the chromosomes. The four interior circles show the distribution of fragments (green), GC islands (orange), repeat regions (black), and SNPs (red) from outside to inside.

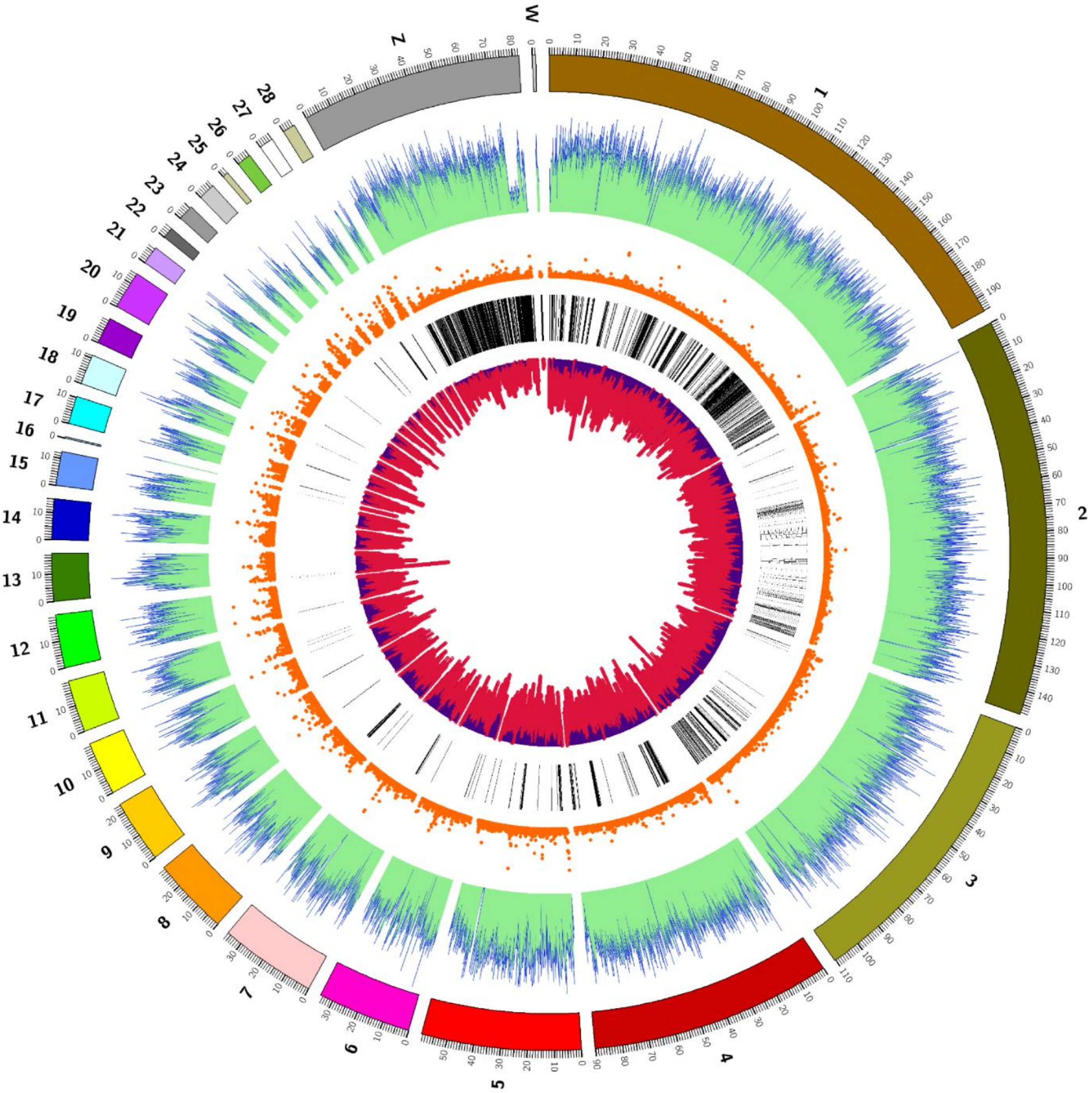

Supplement: S2 Fig — In total, 292 K SNPs were identified among all individuals. The genome characteristics and genome-wide distribution of restricted digest fragments are represented circularly. The exterior circle displays the lengths of the chromosomes. The four interior circles show the distribution of fragments (green), GC islands (orange), repeat regions (black), and SNPs (red) from outside to inside. (PDF) [file pone.0179073.s002.pdf]
