# Supplementary material for: Optimized double-digest genotyping by sequencing (ddGBS) method with high-density SNP markers and high genotyping accuracy for chickens
Source: PLoS One. 2017 Jun 9;12(6):e0179073. doi: 10.1371/journal.pone.0179073 (PMC5466311; doi:10.1371/journal.pone.0179073)
Supplement: S1 Table — (PDF) [file pone.0179073.s003.pdf]

## S1 Table. PCR primers and 24 barcode sequences.

| ID | Sequence (5'to3') | Modify |
|---|---|---|
| GBS_PCR_primerF | AATGATACGGCGACCACCGAGATCTACACTCTTTCCCTACACGACGCTCTTCCGATCT | |
| GBS_PCR_primerR | CAAGCAGAAGACGGCATACGAGATCGGTCTCGGCATTCCTGCTGAACCGCTCTTCCGATCT | |
| CommonAda_Mse_1 | TAAGATCGGAAGAGCGGTTCAGCAGGAATGCCGAG | 5'P |
| CommonAda_Mse_2 | CTCGGCATTCCTGCTGAACCGCTCTTCCGATCT | |
| CommonAda_Msp_1 | CGAGATCGGAAGAGCGGTTCAGCAGGAATGCCGAG | 5'P |
| CommonAda_Msp_2 | CTCGGCATTCCTGCTGAACCGCTCTTCCGATCT | |
| CommonAda_Apek_1 | CWGAGATCGGAAGAGCGGTTCAGCAGGAATGCCGAG | 5'P |
| CommonAda_Apek_2 | CTCGGCATTCCTGCTGAACCGCTCTTCCGATCT | |
| Bar01_Ada_EcoRI_1 | ACACTCTTTCCCTACACGACGCTCTTCCGATCTAACAGT | |
| Bar01_Ada_EcoRI_2 | AATTACTGTTAGATCGGAAGAGCGTCGTGTAGGGAAAGAGTGT | 5'P |
| Bar02_Ada_EcoRI_1 | ACACTCTTTCCCTACACGACGCTCTTCCGATCTCCAGTA | |
| Bar02_Ada_EcoRI_2 | AATTTACTGGAGATCGGAAGAGCGTCGTGTAGGGAAAGAGTGT | 5'P |
| Bar03_Ada_EcoRI_1 | ACACTCTTTCCCTACACGACGCTCTTCCGATCTTTGCAC | |
| Bar03_Ada_EcoRI_2 | AATTGTGCAAAGATCGGAAGAGCGTCGTGTAGGGAAAGAGTGT | 5'P |
| Bar04_Ada_EcoRI_1 | ACACTCTTTCCCTACACGACGCTCTTCCGATCTGGTTCA | |
| Bar04_Ada_EcoRI_2 | AATTTGAACCAGATCGGAAGAGCGTCGTGTAGGGAAAGAGTGT | 5'P |
| Bar05_Ada_EcoRI_1 | ACACTCTTTCCCTACACGACGCTCTTCCGATCTAACGTC | |
| Bar05_Ada_EcoRI_2 | AATTGACGTTAGATCGGAAGAGCGTCGTGTAGGGAAAGAGTGT | 5'P |
| Bar06_Ada_EcoRI_1 | ACACTCTTTCCCTACACGACGCTCTTCCGATCTCCAAGT | |
| Bar06_Ada_EcoRI_2 | AATTACTTGGAGATCGGAAGAGCGTCGTGTAGGGAAAGAGTGT | 5'P |
| Bar07_Ada_PstI_1 | ACACTCTTTCCCTACACGACGCTCTTCCGATCTGAACTGCTGCA | |
| Bar07_Ada_PstI_2 | GCAGTTCAGATCGGAAGAGCGTCGTGTAGGGAAAGAGTGT | 5'P |
| Bar08_Ada_PstI_1 | ACACTCTTTCCCTACACGACGCTCTTCCGATCTACCATGTTGCA | |
| Bar08_Ada_PstI_2 | ACATGGTAGATCGGAAGAGCGTCGTGTAGGGAAAGAGTGT | 5'P |
| Bar09_Ada_PstI_1 | ACACTCTTTCCCTACACGACGCTCTTCCGATCTCTTGAGCTGCA | |
| Bar09_Ada_PstI_2 | GCTCAAGAGATCGGAAGAGCGTCGTGTAGGGAAAGAGTGT | 5'P |
| Bar10_Ada_PstI_1 | ACACTCTTTCCCTACACGACGCTCTTCCGATCTACGTGGTTGCA | |
| Bar10_Ada_PstI_2 | ACCACGTAGATCGGAAGAGCGTCGTGTAGGGAAAGAGTGT | 5'P |
| Bar11_Ada_PstI_1 | ACACTCTTTCCCTACACGACGCTCTTCCGATCTGGAACGTTGCA | |
| Bar11_Ada_PstI_2 | ACGTTCCAGATCGGAAGAGCGTCGTGTAGGGAAAGAGTGT | 5'P |
| Bar12_Ada_PstI_1 | ACACTCTTTCCCTACACGACGCTCTTCCGATCTCACTAGCTGCA | |
| Bar12_Ada_PstI_2 | GCTAGTGAGATCGGAAGAGCGTCGTGTAGGGAAAGAGTGT | 5'P |
| Bar13_Ada_PstI_1 | ACACTCTTTCCCTACACGACGCTCTTCCGATCTATTCTGCTGCA | |
| Bar13_Ada_PstI_2 | GCAGAATAGATCGGAAGAGCGTCGTGTAGGGAAAGAGTGT | 5'P |
| Bar14_Ada_PstI_1 | ACACTCTTTCCCTACACGACGCTCTTCCGATCTCGGAGGTTGCA | |
| Bar14_Ada_PstI_2 | ACCTCCGAGATCGGAAGAGCGTCGTGTAGGGAAAGAGTGT | 5'P |
| Bar15_Ada_PstI_1 | ACACTCTTTCCCTACACGACGCTCTTCCGATCTTAAGAACTGCA | |
| Bar15_Ada_PstI_2 | GTTCTTAAGATCGGAAGAGCGTCGTGTAGGGAAAGAGTGT | 5'P |
| Bar16_Ada_BglII_1 | ACACTCTTTCCCTACACGACGCTCTTCCGATCTGAGAACGT | |

| | | |
|---|---|---|
| Bar16_Ada_BglII_2 | GATCACGTTCTCAGATCGGAAGAGCGTCGTGTAGGGAAAGAGTGT | 5'P |
| Bar17_Ada_BglII_1 | ACACTCTTTCCCTACACGACGCTCTTCCGATCTCCTCCAGC | |
| Bar17_Ada_BglII_2 | GATCGCTGGAGGAGATCGGAAGAGCGTCGTGTAGGGAAAGAGTGT | 5'P |
| Bar18_Ada_BglII_1 | ACACTCTTTCCCTACACGACGCTCTTCCGATCTATCTTGGC | |
| Bar18_Ada_BglII_2 | GATCGCCAAGATAGATCGGAAGAGCGTCGTGTAGGGAAAGAGTGT | 5'P |
| Bar19_Ada_HinpI_1 | ACACTCTTTCCCTACACGACGCTCTTCCGATCTAAGAATCGC | |
| Bar19_Ada_HinpI_2 | CGGCGATTCTTAGATCGGAAGAGCGTCGTGTAGGGAAAGAGTGT | 5'P |
| Bar20_Ada_HinpI_1 | ACACTCTTTCCCTACACGACGCTCTTCCGATCTCCTCCATGC | |
| Bar20_Ada_HinpI_2 | CGGCATGGAGGAGATCGGAAGAGCGTCGTGTAGGGAAAGAGTGT | 5'P |
| Bar21_Ada_HinpI_1 | ACACTCTTTCCCTACACGACGCTCTTCCGATCTATCTTCGGC | |
| Bar21_Ada_HinpI_2 | CGGCCGAAGATAGATCGGAAGAGCGTCGTGTAGGGAAAGAGTGT | 5'P |
| Bar22_Ada_HinpI_1 | ACACTCTTTCCCTACACGACGCTCTTCCGATCTTGGAGGCGC | |
| Bar22_Ada_HinpI_2 | CGGCGCCTCCAAGATCGGAAGAGCGTCGTGTAGGGAAAGAGTGT | 5'P |
| Bar23_Ada_HinpI_1 | ACACTCTTTCCCTACACGACGCTCTTCCGATCTGAAGAATGC | |
| Bar23_Ada_HinpI_2 | CGGCATTCTTCAGATCGGAAGAGCGTCGTGTAGGGAAAGAGTGT | 5'P |
| Bar24_Ada_HinpI_1 | ACACTCTTTCCCTACACGACGCTCTTCCGATCTACCTCTGGC | |
| Bar24_Ada_HinpI_2 | CGGCCAGAGGTAGATCGGAAGAGCGTCGTGTAGGGAAAGAGTGT | 5'P |
