# Supplementary material for: Optimized double-digest genotyping by sequencing (ddGBS) method with high-density SNP markers and high genotyping accuracy for chickens
Source: PLoS One. 2017 Jun 9;12(6):e0179073. doi: 10.1371/journal.pone.0179073 (PMC5466311; doi:10.1371/journal.pone.0179073)
Supplement: S2 Table — (PDF) [file pone.0179073.s004.pdf]

S2 Table. Barcode adaptor (BA) sequences (96-plex).

ID	Sequence (5'to3')	Modify
B01_Ada_EcoRI_1	ACACTCTTTCCCTACACGACGCTCTTCCGATCTAACAGT	
B02_Ada_EcoRI_1	ACACTCTTTCCCTACACGACGCTCTTCCGATCTCCAGTA	
B03_Ada_EcoRI_1	ACACTCTTTCCCTACACGACGCTCTTCCGATCTTTGCAC	
B04_Ada_EcoRI_1	ACACTCTTTCCCTACACGACGCTCTTCCGATCTGCTTCA	
B05_Ada_EcoRI_1	ACACTCTTTCCCTACACGACGCTCTTCCGATCTAACGTC	
B06_Ada_EcoRI_1	ACACTCTTTCCCTACACGACGCTCTTCCGATCTCCAAGT	
B07_Ada_EcoRI_1	ACACTCTTTCCCTACACGACGCTCTTCCGATCTTTGTCA	
B08_Ada_EcoRI_1	ACACTCTTTCCCTACACGACGCTCTTCCGATCTAGTCAC	
B09_Ada_EcoRI_1	ACACTCTTTCCCTACACGACGCTCTTCCGATCTAATGCT	
B10_Ada_EcoRI_1	ACACTCTTTCCCTACACGACGCTCTTCCGATCTCCGATC	
B11_Ada_EcoRI_1	ACACTCTTTCCCTACACGACGCTCTTCCGATCTTTACGA	
B12_Ada_EcoRI_1	ACACTCTTTCCCTACACGACGCTCTTCCGATCTGTCTAT	
B13_Ada_EcoRI_1	ACACTCTTTCCCTACACGACGCTCTTCCGATCTAATCGA	
B14_Ada_EcoRI_1	ACACTCTTTCCCTACACGACGCTCTTCCGATCTCCGTAT	
B15_Ada_EcoRI_1	ACACTCTTTCCCTACACGACGCTCTTCCGATCTTTAGCC	
B16_Ada_EcoRI_1	ACACTCTTTCCCTACACGACGCTCTTCCGATCTCGCATA	
B17_Ada_EcoRI_1	ACACTCTTTCCCTACACGACGCTCTTCCGATCTAAGCAT	
B18_Ada_EcoRI_1	ACACTCTTTCCCTACACGACGCTCTTCCGATCTCTATGC	
B19_Ada_EcoRI_1	ACACTCTTTCCCTACACGACGCTCTTCCGATCTTCCGCA	
B20_Ada_EcoRI_1	ACACTCTTTCCCTACACGACGCTCTTCCGATCTAGTATC	
B21_Ada_EcoRI_1	ACACTCTTTCCCTACACGACGCTCTTCCGATCTGAACCT	
B22_Ada_EcoRI_1	ACACTCTTTCCCTACACGACGCTCTTCCGATCTCCTTGA	
B23_Ada_EcoRI_1	ACACTCTTTCCCTACACGACGCTCTTCCGATCTTTCGAT	
B24_Ada_EcoRI_1	ACACTCTTTCCCTACACGACGCTCTTCCGATCTTGGACC	
B25_Ada_EcoRI_1	ACACTCTTTCCCTACACGACGCTCTTCCGATCTGAACTGC	
B26_Ada_EcoRI_1	ACACTCTTTCCCTACACGACGCTCTTCCGATCTACCATGT	
B27_Ada_EcoRI_1	ACACTCTTTCCCTACACGACGCTCTTCCGATCTCTTGAGC	
B28_Ada_EcoRI_1	ACACTCTTTCCCTACACGACGCTCTTCCGATCTACGTGGT	
B29_Ada_EcoRI_1	ACACTCTTTCCCTACACGACGCTCTTCCGATCTGCAACGT	
B30_Ada_EcoRI_1	ACACTCTTTCCCTACACGACGCTCTTCCGATCTCACTAGC	
B31_Ada_EcoRI_1	ACACTCTTTCCCTACACGACGCTCTTCCGATCTATTCTGC	
B32_Ada_EcoRI_1	ACACTCTTTCCCTACACGACGCTCTTCCGATCTCGGAGGT	
B33_Ada_EcoRI_1	ACACTCTTTCCCTACACGACGCTCTTCCGATCTTAAGAAC	
B34_Ada_EcoRI_1	ACACTCTTTCCCTACACGACGCTCTTCCGATCTGACCTCT	
B35_Ada_EcoRI_1	ACACTCTTTCCCTACACGACGCTCTTCCGATCTACTTCGC	
B36_Ada_EcoRI_1	ACACTCTTTCCCTACACGACGCTCTTCCGATCTCTAGGTT	
B37_Ada_EcoRI_1	ACACTCTTTCCCTACACGACGCTCTTCCGATCTTGGAACC	
B38_Ada_EcoRI_1	ACACTCTTTCCCTACACGACGCTCTTCCGATCTGCCTCAT	
B39_Ada_EcoRI_1	ACACTCTTTCCCTACACGACGCTCTTCCGATCTATTCGTT	
B40_Ada_EcoRI_1	ACACTCTTTCCCTACACGACGCTCTTCCGATCTTAGGTGC	

B41_Ada_EcoRI_1	ACACTCTTTCCCTACACGACGCTCTTCCGATCTCGAATAC	
B42_Ada_EcoRI_1	ACACTCTTTCCCTACACGACGCTCTTCCGATCTCCTCAGT	
B43_Ada_EcoRI_1	ACACTCTTTCCCTACACGACGCTCTTCCGATCTATCTGCC	
B44_Ada_EcoRI_1	ACACTCTTTCCCTACACGACGCTCTTCCGATCTTAGGCTT	
B45_Ada_EcoRI_1	ACACTCTTTCCCTACACGACGCTCTTCCGATCTAGACAAT	
B46_Ada_EcoRI_1	ACACTCTTTCCCTACACGACGCTCTTCCGATCTCCTAGCC	
B47_Ada_EcoRI_1	ACACTCTTTCCCTACACGACGCTCTTCCGATCTATCTCGT	
B48_Ada_EcoRI_1	ACACTCTTTCCCTACACGACGCTCTTCCGATCTTGAGTTC	
B49_Ada_EcoRI_1	ACACTCTTTCCCTACACGACGCTCTTCCGATCTGAGAACGT	
B50_Ada_EcoRI_1	ACACTCTTTCCCTACACGACGCTCTTCCGATCTCCTCCAGC	
B51_Ada_EcoRI_1	ACACTCTTTCCCTACACGACGCTCTTCCGATCTATCTTGGC	
B52_Ada_EcoRI_1	ACACTCTTTCCCTACACGACGCTCTTCCGATCTCGAGGTGT	
B53_Ada_EcoRI_1	ACACTCTTTCCCTACACGACGCTCTTCCGATCTTAGAATGC	
B54_Ada_EcoRI_1	ACACTCTTTCCCTACACGACGCTCTTCCGATCTGCCTTAGT	
B55_Ada_EcoRI_1	ACACTCTTTCCCTACACGACGCTCTTCCGATCTATTCCGGC	
B56_Ada_EcoRI_1	ACACTCTTTCCCTACACGACGCTCTTCCGATCTTGGAGCGT	
B57_Ada_EcoRI_1	ACACTCTTTCCCTACACGACGCTCTTCCGATCTGAAGATGC	
B58_Ada_EcoRI_1	ACACTCTTTCCCTACACGACGCTCTTCCGATCTCCTGTAGT	
B59_Ada_EcoRI_1	ACACTCTTTCCCTACACGACGCTCTTCCGATCTATCCGCGC	
B60_Ada_EcoRI_1	ACACTCTTTCCCTACACGACGCTCTTCCGATCTTAGTCGGT	
B61_Ada_EcoRI_1	ACACTCTTTCCCTACACGACGCTCTTCCGATCTAGAACACT	
B62_Ada_EcoRI_1	ACACTCTTTCCCTACACGACGCTCTTCCGATCTCCTTACGC	
B63_Ada_EcoRI_1	ACACTCTTTCCCTACACGACGCTCTTCCGATCTATCCTGTT	
B64_Ada_EcoRI_1	ACACTCTTTCCCTACACGACGCTCTTCCGATCTTGAGGTCC	
B65_Ada_EcoRI_1	ACACTCTTTCCCTACACGACGCTCTTCCGATCTGAGAAGTC	
B66_Ada_EcoRI_1	ACACTCTTTCCCTACACGACGCTCTTCCGATCTCCGCGAGT	
B67_Ada_EcoRI_1	ACACTCTTTCCCTACACGACGCTCTTCCGATCTATCTCCGT	
B68_Ada_EcoRI_1	ACACTCTTTCCCTACACGACGCTCTTCCGATCTTATGTTCC	
B69_Ada_EcoRI_1	ACACTCTTTCCCTACACGACGCTCTTCCGATCTGTAACTTC	
B70_Ada_EcoRI_1	ACACTCTTTCCCTACACGACGCTCTTCCGATCTCCTCAGCT	
B71_Ada_EcoRI_1	ACACTCTTTCCCTACACGACGCTCTTCCGATCTTTCTGATC	
B72_Ada_EcoRI_1	ACACTCTTTCCCTACACGACGCTCTTCCGATCTGGAGTCGT	
B73_Ada_EcoRI_1	ACACTCTTTCCCTACACGACGCTCTTCCGATCTAAGAATCGC	
B74_Ada_EcoRI_1	ACACTCTTTCCCTACACGACGCTCTTCCGATCTCCTCCATGC	
B75_Ada_EcoRI_1	ACACTCTTTCCCTACACGACGCTCTTCCGATCTATCTTCGGC	
B76_Ada_EcoRI_1	ACACTCTTTCCCTACACGACGCTCTTCCGATCTTGGAGGCGC	
B77_Ada_EcoRI_1	ACACTCTTTCCCTACACGACGCTCTTCCGATCTGAAGAATGC	
B78_Ada_EcoRI_1	ACACTCTTTCCCTACACGACGCTCTTCCGATCTACCTCTGGC	
B79_Ada_EcoRI_1	ACACTCTTTCCCTACACGACGCTCTTCCGATCTCATCTCCGC	
B80_Ada_EcoRI_1	ACACTCTTTCCCTACACGACGCTCTTCCGATCTGTGAGGTGC	
B81_Ada_EcoRI_1	ACACTCTTTCCCTACACGACGCTCTTCCGATCTTGAGAAGGC	
B82_Ada_EcoRI_1	ACACTCTTTCCCTACACGACGCTCTTCCGATCTACCTTGCGC	
B83_Ada_EcoRI_1	ACACTCTTTCCCTACACGACGCTCTTCCGATCTCATCCTGGC	

B84_Ada_EcoRI_1	ACACTCTTTCCCTACACGACGCTCTTCCGATCTTTAGGCTGC	
B85_Ada_EcoRI_1	ACACTCTTTCCCTACACGACGCTCTTCCGATCTCGGAACTCC	
B86_Ada_EcoRI_1	ACACTCTTTCCCTACACGACGCTCTTCCGATCTGCCTCACTC	
B87_Ada_EcoRI_1	ACACTCTTTCCCTACACGACGCTCTTCCGATCTATTCTTGGC	
B88_Ada_EcoRI_1	ACACTCTTTCCCTACACGACGCTCTTCCGATCTTAGAGGTCC	
B89_Ada_EcoRI_1	ACACTCTTTCCCTACACGACGCTCTTCCGATCTAGAGAACTC	
B90_Ada_EcoRI_1	ACACTCTTTCCCTACACGACGCTCTTCCGATCTACTCGCGGC	
B91_Ada_EcoRI_1	ACACTCTTTCCCTACACGACGCTCTTCCGATCTCACTCTCGC	
B92_Ada_EcoRI_1	ACACTCTTTCCCTACACGACGCTCTTCCGATCTTTAGTGTCC	
B93_Ada_EcoRI_1	ACACTCTTTCCCTACACGACGCTCTTCCGATCTCGGAATGTC	
B94_Ada_EcoRI_1	ACACTCTTTCCCTACACGACGCTCTTCCGATCTGCCTTATCC	
B95_Ada_EcoRI_1	ACACTCTTTCCCTACACGACGCTCTTCCGATCTATTCCGCTC	
B96_Ada_EcoRI_1	ACACTCTTTCCCTACACGACGCTCTTCCGATCTTACGGCGGC	
B01_Ada_EcoRI_2	AATTACTGTTAGATCGGAAGAGCGTCGTGTAGGGAAAGAGTGT	5'P
B02_Ada_EcoRI_2	AATTTACTGGAGATCGGAAGAGCGTCGTGTAGGGAAAGAGTGT	5'P
B03_Ada_EcoRI_2	AATTGTGCAAAGATCGGAAGAGCGTCGTGTAGGGAAAGAGTGT	5'P
B04_Ada_EcoRI_2	AATTTGAAGCAGATCGGAAGAGCGTCGTGTAGGGAAAGAGTGT	5'P
B05_Ada_EcoRI_2	AATTGACGTTAGATCGGAAGAGCGTCGTGTAGGGAAAGAGTGT	5'P
B06_Ada_EcoRI_2	AATTACTTGGAGATCGGAAGAGCGTCGTGTAGGGAAAGAGTGT	5'P
B07_Ada_EcoRI_2	AATTTGACAAAGATCGGAAGAGCGTCGTGTAGGGAAAGAGTGT	5'P
B08_Ada_EcoRI_2	AATTGTGACTAGATCGGAAGAGCGTCGTGTAGGGAAAGAGTGT	5'P
B09_Ada_EcoRI_2	AATTAGCATTAGATCGGAAGAGCGTCGTGTAGGGAAAGAGTGT	5'P
B10_Ada_EcoRI_2	AATTGATCGGAGATCGGAAGAGCGTCGTGTAGGGAAAGAGTGT	5'P
B11_Ada_EcoRI_2	AATTTCGTAAAGATCGGAAGAGCGTCGTGTAGGGAAAGAGTGT	5'P
B12_Ada_EcoRI_2	AATTATAGACAGATCGGAAGAGCGTCGTGTAGGGAAAGAGTGT	5'P
B13_Ada_EcoRI_2	AATTTCGATTAGATCGGAAGAGCGTCGTGTAGGGAAAGAGTGT	5'P
B14_Ada_EcoRI_2	AATTATACGGAGATCGGAAGAGCGTCGTGTAGGGAAAGAGTGT	5'P
B15_Ada_EcoRI_2	AATTGGCTAAAGATCGGAAGAGCGTCGTGTAGGGAAAGAGTGT	5'P
B16_Ada_EcoRI_2	AATTTATGCGAGATCGGAAGAGCGTCGTGTAGGGAAAGAGTGT	5'P
B17_Ada_EcoRI_2	AATTATGCTTAGATCGGAAGAGCGTCGTGTAGGGAAAGAGTGT	5'P
B18_Ada_EcoRI_2	AATTGCATAGAGATCGGAAGAGCGTCGTGTAGGGAAAGAGTGT	5'P
B19_Ada_EcoRI_2	AATTTGCGGAAGATCGGAAGAGCGTCGTGTAGGGAAAGAGTGT	5'P
B20_Ada_EcoRI_2	AATTGATACTAGATCGGAAGAGCGTCGTGTAGGGAAAGAGTGT	5'P
B21_Ada_EcoRI_2	AATTAGGTTCAGATCGGAAGAGCGTCGTGTAGGGAAAGAGTGT	5'P
B22_Ada_EcoRI_2	AATTTCAAGGAGATCGGAAGAGCGTCGTGTAGGGAAAGAGTGT	5'P
B23_Ada_EcoRI_2	AATTATCGAAAGATCGGAAGAGCGTCGTGTAGGGAAAGAGTGT	5'P
B24_Ada_EcoRI_2	AATTGGTCCAAGATCGGAAGAGCGTCGTGTAGGGAAAGAGTGT	5'P
B25_Ada_EcoRI_2	AATTGCAGTTCAGATCGGAAGAGCGTCGTGTAGGGAAAGAGTGT	5'P
B26_Ada_EcoRI_2	AATTACATGGTAGATCGGAAGAGCGTCGTGTAGGGAAAGAGTGT	5'P
B27_Ada_EcoRI_2	AATTGCTCAAGAGATCGGAAGAGCGTCGTGTAGGGAAAGAGTGT	5'P
B28_Ada_EcoRI_2	AATTACCACGTAGATCGGAAGAGCGTCGTGTAGGGAAAGAGTGT	5'P
B29_Ada_EcoRI_2	AATTACGTTGCAGATCGGAAGAGCGTCGTGTAGGGAAAGAGTGT	5'P
B30_Ada_EcoRI_2	AATTGCTAGTGAGATCGGAAGAGCGTCGTGTAGGGAAAGAGTGT	5'P

B31_Ada_EcoRI_2	AATTGCAGAATAGATCGGAAGAGCGTCGTGTAGGGAAAGAGTGT	5'P
B32_Ada_EcoRI_2	AATTACCTCCGAGATCGGAAGAGCGTCGTGTAGGGAAAGAGTGT	5'P
B33_Ada_EcoRI_2	AATTGTTCTTAAGATCGGAAGAGCGTCGTGTAGGGAAAGAGTGT	5'P
B34_Ada_EcoRI_2	AATTAGAGGTCAGATCGGAAGAGCGTCGTGTAGGGAAAGAGTGT	5'P
B35_Ada_EcoRI_2	AATTGCGAAGTAGATCGGAAGAGCGTCGTGTAGGGAAAGAGTGT	5'P
B36_Ada_EcoRI_2	AATTAACCTAGAGATCGGAAGAGCGTCGTGTAGGGAAAGAGTGT	5'P
B37_Ada_EcoRI_2	AATTGGTTCCAAGATCGGAAGAGCGTCGTGTAGGGAAAGAGTGT	5'P
B38_Ada_EcoRI_2	AATTATGAGGCAGATCGGAAGAGCGTCGTGTAGGGAAAGAGTGT	5'P
B39_Ada_EcoRI_2	AATTAACGAATAGATCGGAAGAGCGTCGTGTAGGGAAAGAGTGT	5'P
B40_Ada_EcoRI_2	AATTGCACCTAAGATCGGAAGAGCGTCGTGTAGGGAAAGAGTGT	5'P
B41_Ada_EcoRI_2	AATTGTATTCGAGATCGGAAGAGCGTCGTGTAGGGAAAGAGTGT	5'P
B42_Ada_EcoRI_2	AATTACTGAGGAGATCGGAAGAGCGTCGTGTAGGGAAAGAGTGT	5'P
B43_Ada_EcoRI_2	AATTGGCAGATAGATCGGAAGAGCGTCGTGTAGGGAAAGAGTGT	5'P
B44_Ada_EcoRI_2	AATTAAGCCTAAGATCGGAAGAGCGTCGTGTAGGGAAAGAGTGT	5'P
B45_Ada_EcoRI_2	AATTATTGTCTAGATCGGAAGAGCGTCGTGTAGGGAAAGAGTGT	5'P
B46_Ada_EcoRI_2	AATTGGCTAGGAGATCGGAAGAGCGTCGTGTAGGGAAAGAGTGT	5'P
B47_Ada_EcoRI_2	AATTACGAGATAGATCGGAAGAGCGTCGTGTAGGGAAAGAGTGT	5'P
B48_Ada_EcoRI_2	AATTGAACTCAAGATCGGAAGAGCGTCGTGTAGGGAAAGAGTGT	5'P
B49_Ada_EcoRI_2	AATTACGTTCTCAGATCGGAAGAGCGTCGTGTAGGGAAAGAGTGT	5'P
B50_Ada_EcoRI_2	AATTGCTGGAGGAGATCGGAAGAGCGTCGTGTAGGGAAAGAGTGT	5'P
B51_Ada_EcoRI_2	AATTGCCAAGATAGATCGGAAGAGCGTCGTGTAGGGAAAGAGTGT	5'P
B52_Ada_EcoRI_2	AATTACACCTCGAGATCGGAAGAGCGTCGTGTAGGGAAAGAGTGT	5'P
B53_Ada_EcoRI_2	AATTGCATTCTAAGATCGGAAGAGCGTCGTGTAGGGAAAGAGTGT	5'P
B54_Ada_EcoRI_2	AATTACTAAGGCAGATCGGAAGAGCGTCGTGTAGGGAAAGAGTGT	5'P
B55_Ada_EcoRI_2	AATTGCCGGAATAGATCGGAAGAGCGTCGTGTAGGGAAAGAGTGT	5'P
B56_Ada_EcoRI_2	AATTACGCTCCAAGATCGGAAGAGCGTCGTGTAGGGAAAGAGTGT	5'P
B57_Ada_EcoRI_2	AATTGCATCTTCAGATCGGAAGAGCGTCGTGTAGGGAAAGAGTGT	5'P
B58_Ada_EcoRI_2	AATTACTACAGGAGATCGGAAGAGCGTCGTGTAGGGAAAGAGTGT	5'P
B59_Ada_EcoRI_2	AATTGCGCGGATAGATCGGAAGAGCGTCGTGTAGGGAAAGAGTGT	5'P
B60_Ada_EcoRI_2	AATTACCGACTAAGATCGGAAGAGCGTCGTGTAGGGAAAGAGTGT	5'P
B61_Ada_EcoRI_2	AATTAGTGTTCTAGATCGGAAGAGCGTCGTGTAGGGAAAGAGTGT	5'P
B62_Ada_EcoRI_2	AATTGCGTAAGGAGATCGGAAGAGCGTCGTGTAGGGAAAGAGTGT	5'P
B63_Ada_EcoRI_2	AATTAACAGGATAGATCGGAAGAGCGTCGTGTAGGGAAAGAGTGT	5'P
B64_Ada_EcoRI_2	AATTGGACCTCAAGATCGGAAGAGCGTCGTGTAGGGAAAGAGTGT	5'P
B65_Ada_EcoRI_2	AATTGACTTCTCAGATCGGAAGAGCGTCGTGTAGGGAAAGAGTGT	5'P
B66_Ada_EcoRI_2	AATTACTCGCGGAGATCGGAAGAGCGTCGTGTAGGGAAAGAGTGT	5'P
B67_Ada_EcoRI_2	AATTACGGAGATAGATCGGAAGAGCGTCGTGTAGGGAAAGAGTGT	5'P
B68_Ada_EcoRI_2	AATTGGAACATAAGATCGGAAGAGCGTCGTGTAGGGAAAGAGTGT	5'P
B69_Ada_EcoRI_2	AATTGAAGTTACAGATCGGAAGAGCGTCGTGTAGGGAAAGAGTGT	5'P
B70_Ada_EcoRI_2	AATTAGCTGAGGAGATCGGAAGAGCGTCGTGTAGGGAAAGAGTGT	5'P
B71_Ada_EcoRI_2	AATTGATCAGAAAGATCGGAAGAGCGTCGTGTAGGGAAAGAGTGT	5'P
B72_Ada_EcoRI_2	AATTACGACTCCAGATCGGAAGAGCGTCGTGTAGGGAAAGAGTGT	5'P
B73_Ada_EcoRI_2	AATTGCGATTCTTAGATCGGAAGAGCGTCGTGTAGGGAAAGAGTGT	5'P

B74_Ada_EcoRI_2	AATTGCATGGAGGAGATCGGAAGAGCGTCGTGTAGGGAAAGAGTGT	5'P
B75_Ada_EcoRI_2	AATTGCCGAAGATAGATCGGAAGAGCGTCGTGTAGGGAAAGAGTGT	5'P
B76_Ada_EcoRI_2	AATTGCGCCTCCAAGATCGGAAGAGCGTCGTGTAGGGAAAGAGTGT	5'P
B77_Ada_EcoRI_2	AATTGCATTCTTCAGATCGGAAGAGCGTCGTGTAGGGAAAGAGTGT	5'P
B78_Ada_EcoRI_2	AATTGCCAGAGGTAGATCGGAAGAGCGTCGTGTAGGGAAAGAGTGT	5'P
B79_Ada_EcoRI_2	AATTGCGGAGATGAGATCGGAAGAGCGTCGTGTAGGGAAAGAGTGT	5'P
B80_Ada_EcoRI_2	AATTGCACCTCACAGATCGGAAGAGCGTCGTGTAGGGAAAGAGTGT	5'P
B81_Ada_EcoRI_2	AATTGCCTTCTCAAGATCGGAAGAGCGTCGTGTAGGGAAAGAGTGT	5'P
B82_Ada_EcoRI_2	AATTGCGCAAGGTAGATCGGAAGAGCGTCGTGTAGGGAAAGAGTGT	5'P
B83_Ada_EcoRI_2	AATTGCCAGGATGAGATCGGAAGAGCGTCGTGTAGGGAAAGAGTGT	5'P
B84_Ada_EcoRI_2	AATTGCAGCCTAAAGATCGGAAGAGCGTCGTGTAGGGAAAGAGTGT	5'P
B85_Ada_EcoRI_2	AATTGGAGTTCCGAGATCGGAAGAGCGTCGTGTAGGGAAAGAGTGT	5'P
B86_Ada_EcoRI_2	AATTGAGTGAGGCAGATCGGAAGAGCGTCGTGTAGGGAAAGAGTGT	5'P
B87_Ada_EcoRI_2	AATTGCCAAGAATAGATCGGAAGAGCGTCGTGTAGGGAAAGAGTGT	5'P
B88_Ada_EcoRI_2	AATTGGACCTCTAAGATCGGAAGAGCGTCGTGTAGGGAAAGAGTGT	5'P
B89_Ada_EcoRI_2	AATTGAGTTCTCTAGATCGGAAGAGCGTCGTGTAGGGAAAGAGTGT	5'P
B90_Ada_EcoRI_2	AATTGCCGCGAGTAGATCGGAAGAGCGTCGTGTAGGGAAAGAGTGT	5'P
B91_Ada_EcoRI_2	AATTGCGAGAGTGAGATCGGAAGAGCGTCGTGTAGGGAAAGAGTGT	5'P
B92_Ada_EcoRI_2	AATTGGACACTAAAGATCGGAAGAGCGTCGTGTAGGGAAAGAGTGT	5'P
B93_Ada_EcoRI_2	AATTGACATTCCGAGATCGGAAGAGCGTCGTGTAGGGAAAGAGTGT	5'P
B94_Ada_EcoRI_2	AATTGGATAAGGCAGATCGGAAGAGCGTCGTGTAGGGAAAGAGTGT	5'P
B95_Ada_EcoRI_2	AATTGAGCGGAATAGATCGGAAGAGCGTCGTGTAGGGAAAGAGTGT	5'P
B96_Ada_EcoRI_2	AATTGCCGCCGTAAGATCGGAAGAGCGTCGTGTAGGGAAAGAGTGT	5'P
