# Supplementary material for: Optimized double-digest genotyping by sequencing (ddGBS) method with high-density SNP markers and high genotyping accuracy for chickens
Source: PLoS One. 2017 Jun 9;12(6):e0179073. doi: 10.1371/journal.pone.0179073 (PMC5466311; doi:10.1371/journal.pone.0179073)
Supplement: S3 Table — (PDF) [file pone.0179073.s005.pdf]

S3 Table. Number of predicted fragments obtained from seven enzymes and their distribution across the chromosomes.

Chromosome		*Msp* I		*Mse* I		*Pst* I		*Eco*R I		*Bgl* II		*Ape*K I		*Hin*P1 I	
Chr.	Size	Sites	Density (N/Mb)	Sites	Density (N/Mb)	Sites	Density (N/Mb)	Sites	Density (N/Mb)	Sites	Density (N/Mb)	Sites	Density (N/Mb)	Sites	Density (N/Mb)
chr1	195276750	87548	448	1294333	6628	129654	664	53635	275	67025	343	505146	2587	81727	419
chr2	148809762	65509	440	1010713	6792	97855	658	41849	281	51283	345	380751	2559	62452	420
chr3	110447801	50138	454	749246	6784	77380	701	31267	283	38433	348	298925	2706	49189	445
chr4	90216835	44603	494	624900	6927	65519	726	25330	281	30383	337	254311	2819	43642	484
chr5	59580361	36287	609	391339	6568	47306	794	16734	281	20121	338	184314	3094	34732	583
chr6	34951654	22762	651	221531	6338	29626	848	9138	261	11342	325	117337	3357	22228	636
chr7	36245040	22958	633	237666	6557	30078	830	9936	274	12232	337	119572	3299	23284	642
chr8	28767244	21713	755	185824	6460	25102	873	7793	271	9317	324	101814	3539	20186	702
chr9	23441680	18713	798	141736	6046	22894	977	6074	259	7482	319	91338	3896	17897	763
chr10	19911089	17874	898	118926	5973	19451	977	5051	254	6416	322	78914	3963	17777	893
chr11	19401079	15990	824	125077	6447	18071	931	5297	273	6306	325	72451	3734	15802	814
chr12	19897011	17109	860	118333	5947	20596	1035	5018	252	6264	315	81369	4090	16449	827
chr13	17760035	18251	1028	97346	5481	20626	1161	4436	250	5637	317	81449	4586	16664	938
chr14	15161805	17574	1159	81081	5348	18387	1213	3770	249	4720	311	73876	4873	16087	1061
chr15	12656803	13846	1094	66684	5269	15004	1185	3062	242	3895	308	60041	4744	13599	1074
chr16	535270	1044	1950	1057	1975	440	822	106	198	122	228	2107	3936	908	1696
chr17	10454150	15421	1475	47305	4525	15067	1441	2207	211	3110	297	59761	5716	13603	1301
chr18	11219875	14722	1312	54164	4828	14641	1305	2523	225	3366	300	58738	5235	13534	1206
chr19	9983394	13671	1369	48503	4858	13045	1307	2164	217	2915	292	52366	5245	12750	1277
chr20	14302601	16801	1175	71656	5010	17992	1258	3281	229	4301	301	70816	4951	14518	1015
chr21	6802778	9331	1372	31224	4590	8992	1322	1553	228	2015	296	37053	5447	8655	1272
chr22	4081097	4523	1108	20365	4990	5189	1271	956	234	1192	292	21018	5150	4575	1121
chr23	5723239	11418	1995	22048	3852	9248	1616	1040	182	1561	273	38222	6678	10174	1778
chr24	6323281	9309	1472	25811	4082	9880	1562	1207	191	1776	281	39410	6233	8787	1390
chr25	2191139	6218	2838	5447	2486	3301	1507	328	150	497	227	14580	6654	5450	2487
chr26	5329985	10891	2043	18089	3394	9572	1796	890	167	1401	263	38789	7278	9454	1774
chr27	5209285	10400	1996	16463	3160	7370	1415	932	179	1369	263	30764	5906	9261	1778
chr28	4742627	10153	2141	15542	3277	7144	1506	847	179	1177	248	29925	6310	8939	1885
chrZ	82363669	49579	602	514770	6250	60422	734	23016	279	27168	330	235789	2863	46288	562
chrW	1248174	1254	1005	5193	4160	537	430	226	181	385	308	2879	2307	857	687
Total		655610		6362372		820389		269666		333211		3233825		619468	
