# Supplementary material for: Optimized double-digest genotyping by sequencing (ddGBS) method with high-density SNP markers and high genotyping accuracy for chickens
Source: PLoS One. 2017 Jun 9;12(6):e0179073. doi: 10.1371/journal.pone.0179073 (PMC5466311; doi:10.1371/journal.pone.0179073)
Supplement: S4 Table — (PDF) [file pone.0179073.s006.pdf]

S4 Table. Number of genes, SNPs, and novel SNPs discovered from ddGBS, 60K, and 600K SNP chips.

Chromosome			ddGBS			60K Illum.		600K Aff.	
Chr.	Size	Genes	SNPs	Novel SNPs	Density (N/Mb)	SNPs	Density (N/Mb)	SNPs	Density (N/Mb)
1	195,276,750	2,768	58,839	20,747	301	7792	40	106607	546
2	148,809,762	1,926	45,818	15,867	308	5794	39	66908	450
3	110,447,801	1,549	34,272	11,661	310	4464	40	59738	541
4	90,216,835	1,475	28,865	9,671	320	3641	40	45291	502
5	59,580,361	1,191	19,024	6,507	319	2422	41	33047	555
6	34,951,654	640	11,675	3,722	334	1916	55	23295	666
7	36,245,040	653	11,281	3,774	311	1991	55	22790	629
8	28,767,244	633	8,776	3,089	305	1593	55	18685	650
9	23,441,680	532	7,518	2,549	321	1343	57	19590	836
10	19,911,089	515	6,244	2,163	314	1472	74	20756	1042
11	19,401,079	442	5,881	2,170	303	1418	73	15187	783
12	19,897,011	407	5,958	2,071	299	1526	77	15939	801
13	17,760,035	441	5,256	1,937	296	1293	73	12323	694
14	15,161,805	487	4,337	1,533	286	1194	79	14893	982
15	12,656,803	430	3,616	1,388	286	1231	97	12013	949
16	535,270	86	134	76	250	28	52	1048	1958
17	10,454,150	376	2,749	1,157	263	1034	99	10812	1034
18	11,219,875	377	2,913	1,104	260	1016	91	11925	1063
19	9,983,394	383	2,658	1,014	266	1008	101	11047	1107
20	14,302,601	443	3,914	1,499	274	1731	121	10968	767
21	6,802,778	286	1,983	746	291	938	138	10440	1535
22	4,081,097	147	825	391	202	359	88	5101	1250
23	5,723,239	268	1,568	723	274	754	132	7910	1382
24	6,323,281	214	1,533	621	242	873	138	8888	1406
25	2,191,139	255	401	193	183	267	122	3877	1769
26	5,329,985	279	1,123	429	211	790	148	7816	1466
27	5,209,285	334	1,176	539	226	621	119	7087	1360
28	4,742,627	299	1,073	456	226	765	161	7051	1487
Z	82,363,669	1,146	12,313	4,491	149	2069	25	26833	326
W	1,248,174	13	49	16	39	0	0	14	11
Total	1,003,035,513	18,995	291,772	102,304	—	51,343	—	617,879	—
