# Supplementary material for: Optimized double-digest genotyping by sequencing (ddGBS) method with high-density SNP markers and high genotyping accuracy for chickens
Source: PLoS One. 2017 Jun 9;12(6):e0179073. doi: 10.1371/journal.pone.0179073 (PMC5466311; doi:10.1371/journal.pone.0179073)
Supplement: S1 Protocol — (PDF) [file pone.0179073.s007.pdf]

S1 Protocol. Double-digest genotyping by sequencing protocol.

Adapters

1. Suspend single-stranded adapter oligos to 100 μM.
2. Make 50 μL of 10 μM double-stranded adapter: 5 μL oligo1 and 5 μL oligo2, annealing buffer for DNA oligos (5×, Beyotime®, Beijing, China) 10 μL and nuclease-free water 30 μL. Heat to 95 °C and ramp for 25 °C (0.1 °C/s), and hold at 4 °C.
3. Adapter MasterMix (AM): mix barcode adapters (BA) with common adapters (CA) for eight enzyme combinations as follows, and hold at 4 °C. Mixing proportions of barcode adaptors (BA) and common adaptors (CA) were determined according to the numbers of each restriction enzyme fragments *in silico* analysis (S3 Table).

Combination (BA-CA)	BA (μL)	CA (μL)	Nuclease-free Water (μL)	Total (μL)
AM1: *Eco*RI- *Mse*I	0.8	15	84.2	100
AM2: *Eco*RI- *Msp*I	1.6	3.9	194.5	200
AM3: *Pst*I- *Mse*I	2.44	15	82.5	100
AM4: *Pst*I- *Msp*I	2.44	1.95	95.6	100
AM5: *Bgl*II- *Ape*KI	1.84	15	83.1	100
AM6: *Pst*I- *Ape*KI	2.44	9.62	87.9	100
AM7: *Hin*P1I- *Mse*I	1.84	9.62	88.5	100
AM8: *Hin*P1I- *Ape*KI	1	9.62	89.3	100

Normalize DNA

1. DNA was extracted from EDTA-anticoagulated blood using the Qiagen DNeasy Blood and Tissue Kit according to the manufacturer’s instructions (Qiagen, Hilden, Germany).
2. Quantify DNA using Qubit2.0 Fluorometer (Qubit® dsDNA BR Assay Kit) (Thermo, MA, U.S.A.) and prepare DNA plate containing 4 µL of 50 ng/µL DNA (200 ng total).

Restriction

1. Restriction MasterMix (RM) in pre-experiment of eight double-enzyme combinations:

	RM1	RM2	RM3	RM4	RM5	RM6	RM7	RM8
Enzyme I (0.5 µL)	*Eco*RI	*Eco*RI	*Pst*I	*Pst*I	*Bgl*II	*Pst*I	*Hin*P1I	*Hin*P1I
Enzyme II (0.5 µL)	*Mse*I	*Msp*I	*Mse*I	*Msp*I	*Ape*KI	*Ape*KI	*Mse*I	*Ape*KI
Buffer (2 µL)	NEB Cutsmart Buffer					NEB Buffer3.1		
Nuclease-free Water	Up water 13 µL in each MasterMix							

2. Add 16 µL Restriction MasterMix to DNA (4 µL at 50 ng/µL) and mix. Digest at 37 ^{o}C for 12 h, 65 ^{o}C for 30 min, and hold at 4 ^{o}C. Three repeated experiments (three individuals from the F_0 generation) were performed for each combination.
3. Proceed directly to ligation.

Ligation

1. Ligation MasterMix in pre-experiment (eight enzyme combinations × three individuals):

Sample	Plate
T4 DNA ligase (Invitrogen™)	2 μL × 24 = 48 μL
5 x Ligase Reaction Buffer	8 μL × 24 = 192 μL
Nuclease-free Water	5 μL × 24 = 120 μL

2. Cohesive end ligation: add 5 μL Adapter MasterMix (AM1-AM8, respectively) to 20 μL digestion products (cleavage by RM1-RM8, respectively). Add 15 μL Ligation MasterMix, mix well, and spin down.
3. Incubate at 22 °C for 1 h, 65 °C for 30 min, and hold at 4 °C.

Pool, purify, and size-select the library

Note: Purify protocol was referenced by:

https://tools.thermofisher.com/content/sfs/manuals/MAN0009847_IonXpressPlus_gDNA_FragLibraryPrep_UG.pdf

1. Pool 20 μL from each sample ligation to a single tube (240 μL in total). Add 1.3× sample volume of Agencourt® AMPure® XP Reagent (312 μL) to the sample (long fragment was removed in this concentration ratio), pipet up and down five times to thoroughly mix the bead suspension with the DNA, pulse-spin the tube, and incubate the mixture for 15 min at room temperature.
2. Pulse-spin and place the tube on a magnetic rack for 3 min. Carefully remove and discard the supernatant without disturbing the pellet.
3. Without removing the tube from the magnetic rack, add 500 μL of freshly prepared 70% ethanol.

Incubate for 30 s, turning the tube around twice on the magnetic rack to move the beads around. After the solution clears, remove and discard the supernatant without disturbing the pellet.

4. Repeat step 4 for a second wash.
5. To remove residual ethanol, pulse-spin the tube, place it back on the magnetic rack, and carefully remove any remaining supernatant with a 20-μL pipettor without disturbing the pellet.
6. Keeping the tube on the magnetic rack, air-dry the beads at room temperature for 3 min.
7. Remove the tube from the magnetic rack, and add 150 μL of Low TE directly to the pellet to disperse the beads. Pipet the mixture up and down five times, and then vortex the sample for 10 s to mix thoroughly.
8. Pulse-spin and place the tube on the magnetic rack for at least 1 min until the solution clears. Transfer the supernatant containing the eluted DNA to a new 1.5-mL Eppendorf LoBind® Tube without disturbing the pellet.
9. Add the 0.8× sample volume of Agencourt® AMPure® XP Reagent (120 μL) to the sample (short fragment was removed with this concentration ratio), pipet up and down five times to thoroughly mix the bead suspension with the DNA, pulse-spin the tube, and incubate the mixture for 15 min at room temperature. Then, repeat steps 2-6.
10. Remove the tube from the magnetic rack, and add 50 μL of Low TE directly to the pellet to disperse the beads. Pipet the mixture up and down five times, and then vortex the sample for 10 s, to mix thoroughly.
11. Pulse-spin and place the tube on the magnetic rack for at least 3 min until the solution clears. Transfer the supernatant containing the eluted DNA to a new 1.5-mL Eppendorf LoBind® Tube without disturbing the pellet.

PCR amplification

1. Make PCR reactions for the library:

**Reagent**	**1×**
Platinum® PCR SuperMix, High Fidelity	50 μL
Library DNA (purified and size-selected)	10 ng
GBS_PCR_primerF (20 μM)	1.2 μL
GBS_PCR_primerR (20 μM)	1.2 μL
UP Nuclease-free water	? μL
**Total**	60 μL

2. Program: 95 ℃ for 5 min; 95 ℃ for 30 s, 62 ℃ for 30 s, and 68 ℃ for 30 s for 17 cycles; followed by a final extension at 72 ℃ for 5 min, and hold at 4 °C.
3. The PCR product was purified according to the "purify and size-selected the library" step.

Library quality control

Final library quality (concentration and fragment size distribution) was controlled by Qubit2.0 Fluorometer (Qubit® dsDNA BR Assay Kit) (Thermo, MA, U.S.A.) and Agilent 2100 Bioanalyzer, respectively(Agilent, Santa Clara, CA, U.S.A.).
